# Experimental evidence of effective human–AI collaboration in medical decision-making

**DOI:** 10.1038/s41598-022-18751-2

**Published:** 2022-09-02

**Authors:** Carlo Reverberi, Tommaso Rigon, Aldo Solari, Cesare Hassan, Paolo Cherubini, GI Genius CADx Study Group, Giulio Antonelli, Halim Awadie, Sebastian Bernhofer, Sabela Carballal, Mário Dinis-Ribeiro, Agnès Fernández-Clotett, Glòria Fernández Esparrach, Ian Gralnek, Yuta Higasa, Taku Hirabayashi, Tatsuki Hirai, Mineo Iwatate, Miki Kawano, Markus Mader, Andreas Maieron, Sebastian Mattes, Tastuya Nakai, Ingrid Ordas, Raquel Ortigão, Oswaldo Ortiz Zúñiga, Maria Pellisé, Cláudia Pinto, Florian Riedl, Ariadna Sánchez, Emanuel Steiner, Yukari Tanaka, Andrea Cherubini

**Affiliations:** 1 Department of Psychology, University of Milano-Bicocca, 20126 Milan, Italy; 2 Milan Center for Neuroscience, University of Milano-Bicocca, 20126 Milan, Italy; 3 Department of Economics, Management and Statistics, University of Milano-Bicocca, 20126 Milan, Italy; 4 Department of Biomedical Sciences, Humanitas University, 20072 Pieve Emanuele, Italy; 5 Endoscopy Unit, Humanitas Clinical and Research Center IRCCS, Rozzano, Italy; 6 Department of Neural and Behavioral Sciences, University of Pavia, Pavia, Italy; 7 Artificial Intelligence Group, Cosmo AI/Linkverse, Lainate, 20045 Milan, Italy; 8 Gastroenterology and Digestive Endoscopy Unit, Ospedale dei Castelli (N.O.C.), Ariccia, Italy; 9 Gastrointestinal and Liver Institute, Emek Medical Center, Afula, Israel; 10 Gastroenterology and Hepatology and Rheumatology, University Hospital of St. Pölten, St. Pölten, Austria; 11 Gastroenterology Department, Hospital Clinic of Barcelona, Barcelona, Spain; 12 Gastroenterology Department, Portuguese Oncology Institute of Porto, Porto, Portugal; 13 Department of Gastroenterology, Kita-Harima Medical Center, Ono, Japan; 14 Gastroenterology Department, Hyogo Cancer Center, Hyogo, Japan; 15 Gastroenterology Department, Sugita Genpaku Memorial Obama Municipal Hospital, Obama, Japan; 16 Gastrointestinal Center, Sano Hospital, Hyogo, Japan; 17 Kobe Red Cross Hospital, Hyogo, Japan; 18 Kobe University Hospital, Hyogo, Japan

**Keywords:** Diagnosis, Colonoscopy, Human behaviour, Machine learning

## Abstract

Artificial Intelligence (ai) systems are precious support for decision-making, with many applications also in the medical domain. The interaction between mds and ai enjoys a renewed interest following the increased possibilities of deep learning devices. However, we still have limited evidence-based knowledge of the context, design, and psychological mechanisms that craft an optimal human–ai collaboration. In this multicentric study, 21 endoscopists reviewed 504 videos of lesions prospectively acquired from real colonoscopies. They were asked to provide an optical diagnosis with and without the assistance of an ai support system. Endoscopists were influenced by ai ($\textsc {or}=3.05$), but not erratically: they followed the ai advice more when it was correct ($\textsc {or}=3.48$) than incorrect ($\textsc {or}=1.85$). Endoscopists achieved this outcome through a weighted integration of their and the ai opinions, considering the case-by-case estimations of the two reliabilities. This Bayesian-like rational behavior allowed the human–ai hybrid team to outperform both agents taken alone. We discuss the features of the human–ai interaction that determined this favorable outcome.

## Introduction

Artificial Intelligence systems are increasingly recognized as a precious tool for improving medical-decision making^1^. ai may support Medical Doctors (mds) in multiple domains (with applications in dermatology, ophthalmology, cardiology, gastroenterology, and mental health, among others) while typically mds keep the final decision. Such complementarity advises a collaboration between human and artificial minds, fostering a “hybrid intelligence” that could deliver outcomes superior to those reached by each mind alone^2,3^. Pivotal to fulfilling this promise is improving the interaction between humans and machines to build up an effective team avoiding pitfalls such as:

1. *over-reliance*: mds adhere to whatever opinion is offered by the ai, ignoring their independent evaluation. This attitude throws away all the information embedded in the md’s own opinion and could endanger the accuracy of the final diagnosis. Previous studies on human trust toward ai decision-support systems alerted us of the possibility of an extreme form of over-reliance, termed “automation bias” for ai’s false alarms and “automation complacency” for ai’s false reassurances. In both cases, the humans uncritically adhere to the machine’s output, ignoring their independent evaluation^4–6^.
2. *under-reliance*: mds display limited trust in the machine and mostly ignore its suggestions, even when informative. If this attitude were dominant, ai would prove useless to any practical means. The extreme form of under-reliance is termed “algorithm aversion”^4,7,8^: the human does not trust the machine and completely ignores its suggestions.
3. *opacity* of judgments’ reliability: in this case, even if mds have an appropriate level of trust towards ai, they cannot tell whether ai opinions are more or less reliable than their own. Opacity may prevent md from reaching an optimal use of the information provided by the ai. Previous studies have addressed this topic by trying to convey the ai’s internal motives of ai decisions, but with mixed results^6,9^.

**Figure 1:**
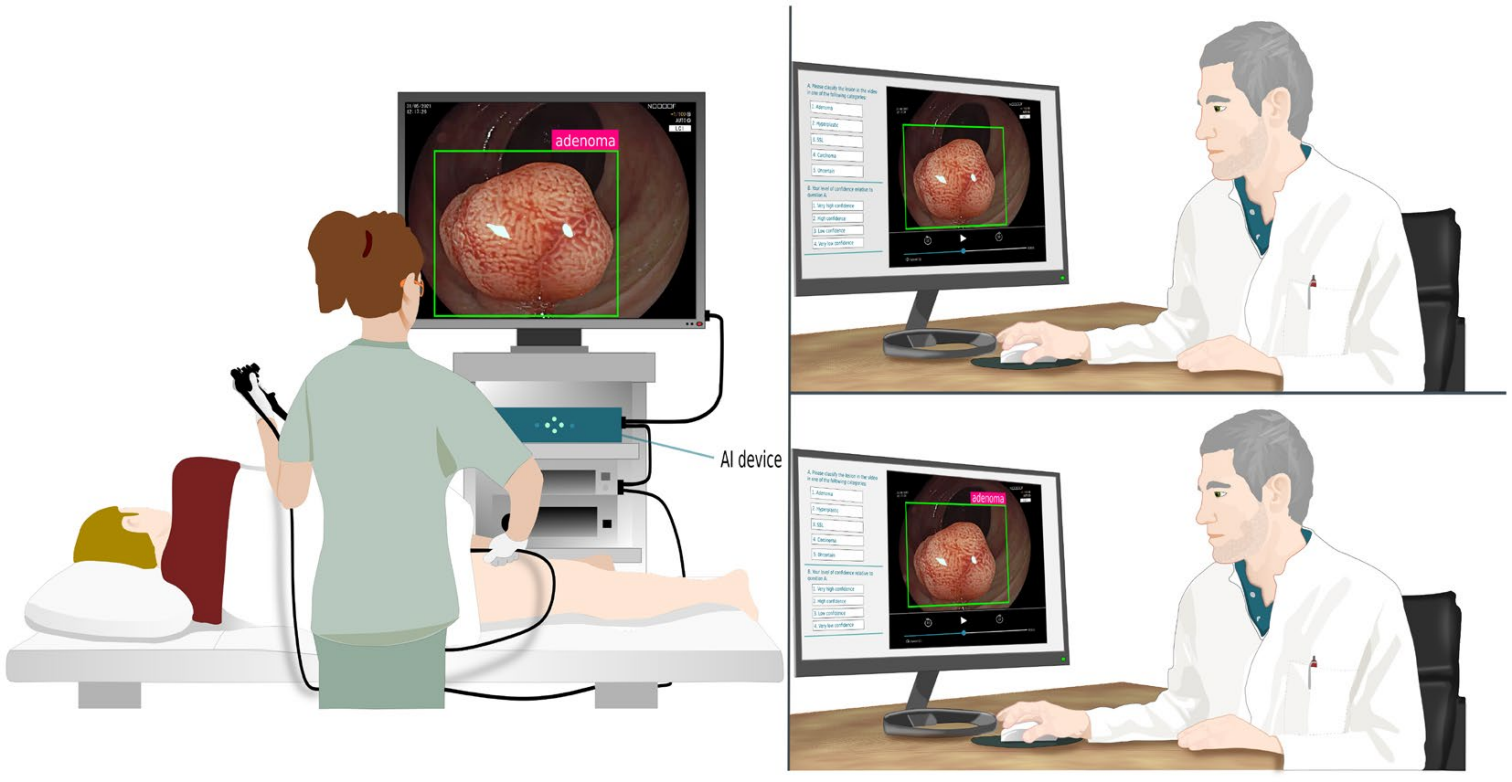
Left panel: The stimuli used in the experiment were prospectively collected in a real-world clinical setting using an ai medical device supporting mds for lesion detection (cade) and categorization (cadx) as adenomatous or non-adenomatous^24^. Right panel: An international group of endoscopists were asked to optically diagnose the same set of lesions, presented as short video clips, in two experimental sessions. In the first session (top-right panel) the ai only highlights the target lesion, while in the second session (bottom-right panel) ai also dynamically offers an optical diagnosis. For more details on the ai device see Appendix A.1.2.

The success and the potential drawbacks of human–ai collaboration are under active scrutiny in the clinical domain and beyond. A renewed interest on this topic followed the new possibilities granted by deep learning tools, thus generating several related lines of research (e.g. “augmented intelligence”, “hybrid intelligence”, “human–ai collaboration”, “human–ai teaming”)^2,3,10–14^. Notwithstanding a clear agenda on the issues that we should explore further, we still have limited evidence-based knowledge of the context, design, and psychological mechanisms that would craft an optimal human–ai team^15–17^. Many experimental studies and reviews measured the performance of ai-based medical devices, or the improvement of the mds diagnostic accuracy when supported by ai (e.g.,^18–22^). One review focused specifically on radiologists trust in ai’s recommendations^23^. To our knowledge, no previous experimental study addressed the inner dynamics of ai-supported mds’ beliefs revision. ai assisted colonoscopy provides a privileged case study on team-working between humans and machines. The colonoscopic procedure naturally emphasizes the complementary roles of the endoscopist and the ai (Fig. 1). On one side, the endoscopist is fundamental for navigating the probe and selecting the information. On the other hand, the limited time available combined with the intense multitasking and the multidimensional nature of lesion classification imply the potential benefit of additional help.

**Figure 2:**
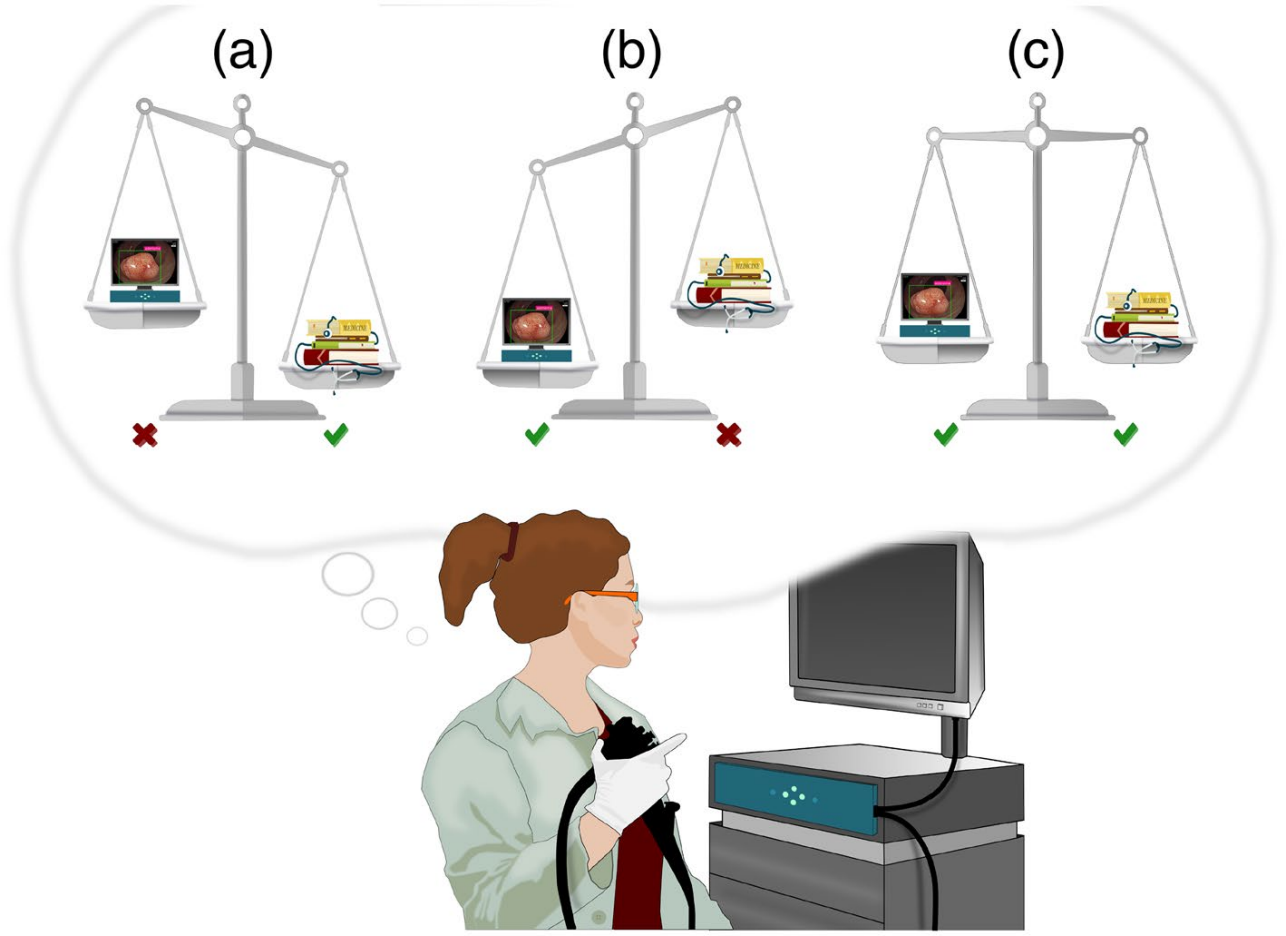
md-ai team. An endoscopist subject to under-reliance discounts the added information given by the ai (**a**). An endoscopist subject to over-reliance supinely accepts the ai suggestion (**b**). The optimal use of ai should rest on an in-between, well-calibrated approach where the endoscopist uses the ai opinion for coherently revising their confidence in their initial evaluation. In this way, the medical decision-making process would benefit from a collaboration between the two intelligences (**c**).

We study the interaction of endoscopists and a last-generation decision support system^24^ during the optical examination of colorectal lesions. We model the endoscopist diagnosis as a psychological categorization process^25,26^. Incoming visual information is compared to previously stored knowledge about a finite set of possible diagnoses in a Bayesian-like procedure that revises the endoscopist’s confidence toward those diagnoses. Accrued information can make one diagnosis dominant over its alternatives. We aim to understand how the availability of the ai advice influences the opinion of the endoscopist (i.e., whether an “effective hybrid team” is formed) and whether individual features like the endoscopist expertise modulate the ai influence. In qualitative Bayesian terms, the ai output is a further piece of information that should be integrated to revise the endoscopist’s diagnosis, not differently as it would happen by considering an “informed opinion” volunteered by a human colleague with a slightly different expertise profile. If such well-calibrated interaction is achieved, endoscopists’ accuracy with ai should improve, notwithstanding their baseline level of accuracy without ai, because they would have available one further piece of information (Fig. 2).

Expert and novice endoscopists were asked to optically diagnose colorectal lesions. The same set of lesions ($n=504$) was presented, as short video clips, in two experimental sessions. In the first session the ai (Fig. 1) only highlights the target lesion, while in the second session ai also dynamically offers an optical diagnosis. We had four leading experimental questions, i.e., whether endoscopists are influenced by the ai opinion, and in case, whether this leads to an improvement of diagnostic accuracy; whether the endoscopists could selectively follow the ai when it is correct and conversely reject ai’s opinion when it is incorrect. Overall, we hypothesized that endoscopists consider ai’s opinion to improve their diagnostic performance and can discriminate correct from potentially wrong ai’s opinions. Further planned measures and analyses aimed at clarifying the reasons underlying the endoscopists’ behavior and the interaction between humans and ai. Namely, we hypothesized that endoscopists have a reliable insight on the correctness likelihood of their own and ai’s opinions and that they use such insight to weight the human and ai judgment. Finally, a larger increase in accuracy should be observed for the non-expert endoscopists. Our predictions were pre-registered before the data gathering, together with the study plan, the statistical models, and the analyses (preregistration is available at https://osf.io/y9at5).

Our study is an original contribution to the literature in several ways. First, we investigated whether and how the output of an ai real-time classifier influences the decision of a md in a within-subject design that compares decisions of the same md, with and without AI support, on a prospectively acquired dataset of colonoscopy videos. Previous studies on cadx systems for colonoscopy focused on assessing the performances of ai against the accuracy of physicians^19,20,27–30^, or against the criteria established by gastroenterology societies for implementing the technology in a clinical setting^31–34^.

Second, we developed a new, rigorous statistical model for measuring mds’ belief revisions in experimental settings by framing the mds’ diagnostic updates following ai advice as a Bayesian-like belief-revision process. The model separates “efficacy” (whether the md aligns her belief to the ai when the latter is correct) and “safety” (whether the md stays with her previous belief when the ai is wrong). Thus the model transparently assesses the positive and negative impact of ai opinions on md decisions and their ability to avoid over/under reliance.

Third, and importantly, we explored the psychological processes underlying the emergence of an effective hybrid team, even when humans need to interact with a non-transparent ai device (i.e., a device not conveying the motives of its decisions). For that aim, we collected the critical parameters that should contribute to a final mds decision: the mds opinion, their confidence, their interpretation of the ai output, and the perceived reliability of each ai advice. These parameters were ignored in previous studies on ai medical devices.

## Methods

### Study design and participants

This is an experimental study with a mixed $2 \times 2$ design, in which the within-subjects factor is the treatment (no ai vs. ai), and the between-subjects factor is the endoscopists level of expertise (experts vs. non-experts). Twenty-one endoscopists took part in the experiment, 10 of them experts (at least 5 years of colonoscopy experience and experience in optical biopsy with virtual chromoendoscopy) and 11 non-expert (less than 500 colonoscopies performed). The acquired sample size ensures adequate statistical power for testing our main experimental endpoints (the power analysis is in Appendix A.5). The participants were from Austria, Israel, Japan, Portugal, and Spain (see the consortium members at the end of the paper). Both the task and task instructions were in English. All methods were carried out, and results were reported following STROBE guidelines and regulations. The study was approved by the local Institutional Review Board (Comitato Etico Lazio 1, prot. 611/CE Lazio1) and conducted following the Declaration of Helsinki. All participants provided written informed consent.

### Procedure and data collection

The experiment is divided into two sessions. In *Session 1 (*S1 ), endoscopists diagnose the lesions without ai advice. Endoscopists watched on an online dedicated platform 504 videos of real colonoscopies, each presenting one lesion. Endoscopists examined lesions at their own pace. Their task was, first, to categorize each lesion in five forced-choice options: “Adenoma”, “Hyperplastic”, “ssl”, “Carcinoma”, “Uncertain”. Their choices were later mapped to the 3-fold output “Adenoma” (corresponding to the choices “Adenoma” or “Carcinoma”), “Non-Adenoma” (for “Hyperplastic” or “ssl” choices), or “Uncertain”. The second task was to describe their confidence in their decision as: “Very high”, “High”, “Low”, or “Very low”. We recorded the time elapsed from the beginning of the video to the first decision. No feedback was provided. In *Session 2* (S2), endoscopists’ decision was supported by ai: the endoscopists saw the same videos as in S1, but with the ai’s dynamic advice for each lesion: the ai’s optical diagnosis for a specific lesion could vary between frames, displaying one of the four possible values “adenoma”, “non-adenoma”, “no-prediction” or “analyzing” (see Appendix A.1.2). The task and lesions were the same as in S1. However, besides the two questions identical to S1, in S2 the endoscopists had to also report the perceived ai’s overall opinion about the lesion, using 3 forced-choice response options: “Adenoma”, “Non-Adenoma”, and “Uncertain”; and the perceived ai’s level of confidence about the lesion, using 4 forced-choice response options: “Very high”, “High”, “Low”, and “Very low”. The 504 videos in each session were divided into 6 batches of 84 videos each, with a predefined sequence of administration of the batches. For each batch, we prepared different pre-randomized orders of presentation of the lesions. Each participant was preassigned to a different order. At least two weeks passed from the conclusion of the evaluation of one batch in S1 and the evaluation of the same batch in S2 to avoid memory effects. All stimuli were acquired in full length and with no ai overlay in the *CHANGE* clinical study^34^. For generating S1 clips, we used GI Genius v3.0 in cade modality to dynamically add a green box around the suspect lesions automatically detected by the device (see Appendix A.1.2 for a detailed description of the ai system). For S2 we used GI Genius v3.0 in cade+cadx modality^24^, dynamically overlaying a green box around suspected lesions and the optical diagnosis computed by the ai. For evaluating human and AI performance, we considered the histopathological diagnosis of each lesion as ground truth. A more detailed description of stimuli and procedure is in the Appendix A. Three example stimuli are available online; for a description of the video clips, see Appendix A.1.1. The recorded and transformed variables are described in Table 1.

**Table 1:** Measured and transformed variables for each of the 21 subjects and 504 lesions.

Variable name	Description
Histologic evaluation	The ground truth of each lesion. Its possible values were: “Adenoma”, “Non-Adenoma”
Human judgment, S1 and S2	The optical diagnosis of the lesion by an endoscopist in each session, mapped as mentioned above. It takes the values: “Adenoma”, ‘Non-adenoma”, and “Uncertain”
Human confidence, S1 and S2	The confidence of the previous judgment expressed by the endoscopist. It takes the values: “Very high”, “High”, “Low”, “Very low”, “Uncertain”. We classified the confidence as “Uncertain” whenever the associated lesion evaluation was “Uncertain”
Algorithmic ai diagnosis	The diagnosis about a given lesion provided by the ai output in S2, as interpreted by an automatic algorithm. It takes the values: “Adenoma”, “Non-Adenoma”, and “Undetermined”
Perceived ai diagnosis	The endoscopists’ interpretation of the ai dynamic output in S2. It takes the following values: “Adenoma”, “Non-Adenoma”, “Uncertain”, and “I am not sure/I did not notice the output of the ai”
Evaluation of ai confidence	The endoscopists’ appreciation of the level of reliability of the ai diagnosis in S2. It takes the following values: “Very high confidence”, “High confidence”, “Low confidence”, and “Very low confidence”
**Transformed variable name**	
Human correct diagnosis, S1 and S2	A binary variable that indicates whether each lesion was correctly diagnosed by each endoscopist, in each session
Accuracy$_{s}$ of the perceived ai diagnosis	A binary variable indicating whether each lesion is correctly diagnosed by the ai. We considered the ai diagnosis perceived by the endoscopist. “Uncertain” and “not sure/not notice” were excluded from the computation
Accuracy$_{u}$ of the perceived ai diagnosis	A binary variable indicating whether each lesion is correctly diagnosed by the ai. We considered the ai diagnosis judged by the endoscopist. Classifications of the ai output as “Uncertain” and “not sure/not notice” were conservatively considered errors
Confidence score, S1 and S2	A discrete numerical variable ranging 1 to 9 that measures the belief of each endoscopist about each lesion in each session. The score of 9 indicates a strong belief that the lesion is an adenoma. At the other extreme, the score of 1 denotes a strong belief that the lesion is not an adenoma. The score of 5 indicates “Uncertain” diagnoses

### Statistical analyses

For comparing the probability of prediction-relevant events during S1 with the probability of the same events during S2, for each endoscopist, we defined and computed four odds ratios amenable to be analyzed by a logistic regression statistical model (see Appendix A.4). The four main experimental endpoints and the related odds ratios are:

1. ai *influence* on endoscopists’ decision ($\omega _I$): the ratio between the odds that the endoscopists’ diagnoses were the same as ai’s diagnoses in S2, and the odds that the endoscopists’ diagnoses in S1 were the same as those given by ai on the same lesions in S2, irrespective to accuracy. Values greater than 1 mean that in S2 the endoscopists’ diagnoses converged on ai diagnoses. The opinion of the endoscopist should be influenced by the response of the ai, i.e., we hypothesize $\omega _I > 1$.
2. ai *effect on diagnostic accuracy* ($\omega _A$): computed as a ratio between the odds that the endoscopists’ diagnoses were correct in S2, and the odds that the endoscopists’ diagnoses were correct in S1. Values greater than 1 mean that ai’s assistance is associated with an increased probability of a correct human diagnosis. Accuracy should improve with ai, thus $\omega _A > 1$.
3. *Effectiveness* ($\omega _E$): same as $\omega _A$, but using odds estimated only on the subset of lesions where ai returned a correct diagnosis. Values greater than 1 mean that when ai is correct, ai’s assistance increases the probability of a correct human diagnosis. The endoscopists should rightfully accept the ai opinion when this is correct, thus $\omega _E > 1$.
4. *Safety* ($\omega _S$): same as $\omega _A$, but using odds estimated only on the subset of lesions where ai returned a wrong diagnosis. Values less than 1 mean that when ai is incorrect, ai’s assistance deteriorates the endoscopists’ diagnostic performance. The endoscopists should be able to disengage from a wrong opinion of the ai so that their S1 performance is not remarkably deteriorated when ai’s offers a wrong suggestion. Thus, we hypothesized $\omega _S > 0.3$.

The above four sets of odds ratios $\omega _I, \omega _A, \omega _E, \omega _S$ are obtained as the result of logistic regression models that account for lesion- and endoscopist- specific random effects. The parameters and the corresponding odds ratios of the logistic regressions were estimated via (integrated) maximum likelihood and are based on the lme4^35^. Related inferential quantities (e.g., confidence intervals) were also computed using the lme4 R package. The complete mathematical definitions of the transformed variables, the odds ratios, the relative risks, the statistical models used, and the details of the inferential tests run are available online in Appendix A.4, A.3, and A.6.

**Table 2:** Odds-ratios (or) for each of the main endpoints. We report in brackets the $95\%$ confidence intervals for the odds ratios.

Endpoint	or	Estimate
1. Influence of the ai	$\omega _I$	3.05 [2.76, 3.39]
2. Diagnostic accuracy	$\omega _A$	1.39 [1.28, 1.51]
3. Effectiveness	$\omega _E$	3.48 [3.07, 3.98]
4. Safety	$\omega _S$	0.54 [0.48, 0.62]

## Results

**Figure 3:**
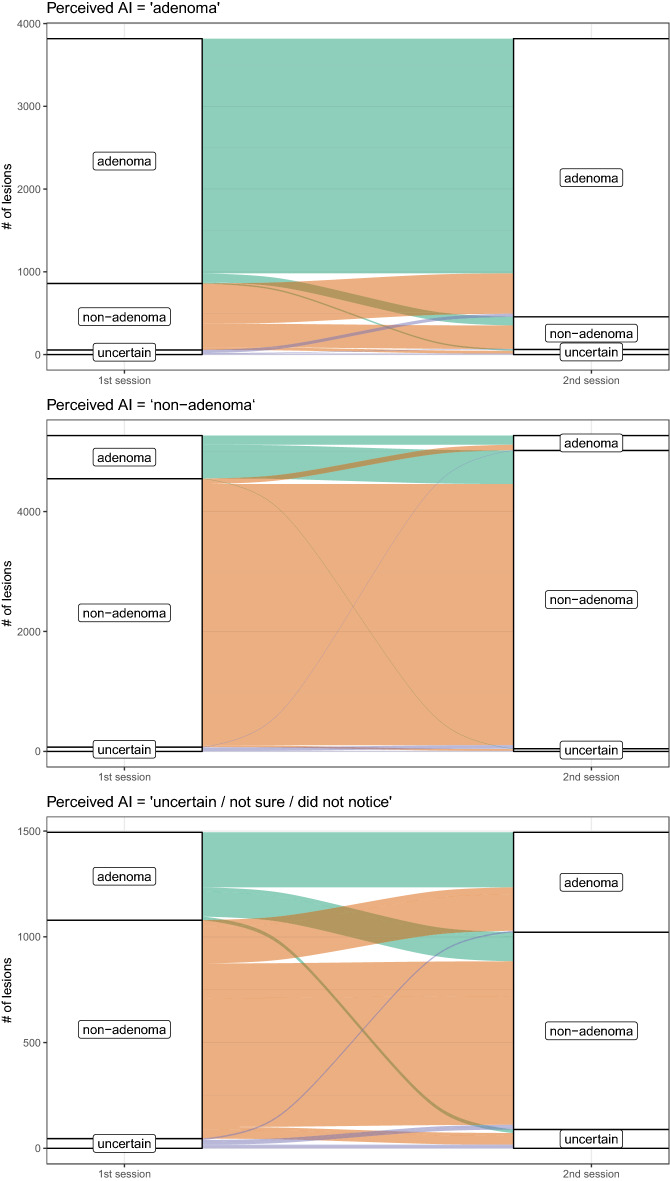
Influence of the ai: alluvial diagrams representing changes in endoscopist’s opinion between the two sessions as a function of perceived ai response.

Our main, pre-registered expectations were fully supported by results (Table 2, Fig. 3, see section “Statistical analyses” for details on the measures used). Endoscopists were influenced by the ai opinion ($\omega _I=3.05$). On average, for every three lesions over which endoscopists disagreed with ai in S1, only one remained in S2. Considering ai opinion is beneficial to the diagnostic performance of the endoscopists: every five lesions correctly evaluated without ai, seven were correctly evaluated with ai. Importantly, endoscopists could discriminate the good from the bad ai advice. When the ai was correct, the endoscopists followed its advice more ($\omega _E=3.48$) than when the ai was incorrect ($1/\omega _S=1.85$). In other words, endoscopists could not fully escape from the negative consequences of a wrong ai advice (i.e., $\omega _S$ is less than 1), but this was more than compensated by their stronger tendency to accept a correct ai advice.

### Additional analyses: explaining endoscopist behavior

Overall, additional analyses aimed at understanding the reasons underlying the observed endoscopists’ behavior. We group additional results into two sets: endoscopists’ assessment of the main task parameters, ai and expertise effects. Further results (e.g., time to decision, individual-level performance) and alternative analysis approaches (e.g., roc curves) are reported in Appendix B.

#### Endoscopists’ assessment of the main task parameters

We hypothesized that the endoscopists are aware of the changing soundness of their judgments and are naively able to interpret the ai output and assess its reliability. To test these hypotheses, we considered three measures: the confidence of the endoscopists over their diagnosis, the endoscopists’ interpretation of the ai output, and its confidence. We found that endoscopists’ confidence in an optical diagnosis is strongly predictive of its accuracy in both sessions: the higher the confidence, the higher the accuracy (Table 3). The endoscopists’ interpretation of the ai output is overall consistent with an algorithmic assessment of the ai output (81% of agreement between endoscopists and ai, 94% agreement when judgments “uncertain” are excluded). It is consistent across endoscopists (77% of average agreement among all the possible pairs of endoscopists, 94% when “uncertain” are excluded). Finally, and importantly, the endoscopists’ estimates of the ai reliability are predictive of ai accuracy (Table 3).

Having shown that endoscopists can generate meaningful estimates of critical decision parameters, we asked whether they used them to optimally integrate their opinion with that of ai. An intuitive Bayesian decision-maker would weigh each opinion over its estimated reliability. We thus asked whether the influence of ai on endoscopists’ decisions would change depending on their own and ai confidence. We found that this is indeed the case: when endoscopist confidence (as estimated in S1) is high, or ai confidence is low, the endoscopists tend to stick with their decision, i.e., they do not change their mind in case of disagreement with ai (Table 4). The other way around in case of low endoscopist confidence or high ai confidence. Similar results were obtained in a related analysis on confidence scores (see Appendix B.3).

**Table 3:** Proportions and sample sizes of the correct human diagnosis in S1, S2 and the ai perceived correct diagnosis, against different human confidence levels and the ai perceived confidence levels, respectively. Following the standard in the field, accuracy_s_ does not consider wrong lesions where ai opinion was perceived as “uncertain” or was “not noticed”. Evaluations of the confidence of the ai were asked only when the opinion was “Adenoma” or “Non-Adenoma”.

Confidence	Very low	Low	High	Very high	Overall
S1 accuracy	0.644 (236)	0.685 (2665)	0.806 (5263)	0.853 (2241)	0.768 (10,584)
S2 accuracy	0.543 (184)	0.679 (1859)	0.839 (5235)	0.882 (3094)	0.802 (10,584)
ai accuracy_s_	0.667 (216)	0.718 (1456)	0.863 (4608)	0.909 (2807)	0.849 (9086)

**Table 4:** Change in *agreement* between endoscopists’ and ai, measured as the amount of times each endoscopist changes its opinion and follows ai’s suggestion. We report proportions and sample sizes of the change in *agreement* for different human confidence levels (S1) and the ai perceived confidence levels, respectively.

	Very low	Low	High	Very high
Human conf.	0.703 (64)	0.738 (577)	0.668 (689)	0.598 (194)
ai conf.	0.278 (97)	0.438 (457)	0.827 (684)	0.888 (286)

#### Effects of expertise

Endoscopists’ expertise modulated the variables considered for our main analyses. ai affected non-experts more than experts, and it had a larger impact on accuracy, possibly because experts’ had less to gain from ai’s added information since their accuracy was close to the ai accuracy. Furthermore, experts were less able than non-experts to discriminate between good and bad ai advice: both efficacy and safety are higher in non-experts than in experts (Table 5). The lower safety of experts seems at first counter-intuitive. However, it may be understood as a stronger preference of experts to avoid false-negative errors at the cost of increasing false positives: experts accept more than non-experts an incorrect “adenoma” advice from ai. This is arguably a clinically prudent approach in colonoscopies. The interpretation is supported by the observation that in S2 non-experts increased both their specificity and sensitivity, whereas experts increased only their sensitivity (see Appendix B.5).

The average confidence of experts was lower than those of non-experts, in all sessions, both towards their own judgments and towards ai output. However, no differences were present in the relative trust towards ai in the two groups: both groups had a slightly higher average confidence towards ai as compared to themselves (see Fig. B.11 in Appendix). This observation implies that confidence cannot explain the different attitudes of experts towards ai. More importantly, the confidence of both non-experts and experts was predictive of real accuracy (own or ai). This finding means that both expertise groups can appropriately use confidence to inform their final decision.

**Table 5:** Odds-ratios (or) for each endpoint, estimated separately for experts and non experts. We report in brackets the $95\%$ confidence intervals.

Endpoint	or	Expert	Non-expert
1. Influence of the ai	$\omega _I$	2.88 [2.48, 3.34]	3.20 [2.80, 3.65]
2. Diagnostic accuracy	$\omega _A$	1.15 [1.01, 1.30]	1.61 [1.44, 1.79]
3. Effectiveness	$\omega _E$	3.22 [2.64, 3.93]	3.65 [3.11, 4.28]
4. Safety	$\omega _S$	0.45 [0.37, 0.54]	0.63 [0.54, 0.75]

### ai performance

Depending on how one interprets the rich, dynamic output of the ai, the ai accuracy would change. To provide a fuller picture, we report the ai accuracy in multiple ways. First, we considered the human interpretation of the ai output: the ai standard accuracy (accuracy_s__*s*_), which excludes “uncertain” or “not sure/not notice” outputs from consideration, is 84.9%. A more conservative accuracy that includes also “uncertain” and “not sure/not notice” outputs as errors (accuracy_*u*_) is 72.9%. On average, we observed 71 ai outputs classified by the endoscopists as “uncertain” or “not sure/not notice” out of 504. The ai accuracy based on human interpretations varies across individuals: see Appendix B.2 for details. Second, when an automated algorithm interprets the output, the ai accuracy is 84.5% when the label “uncertain” is excluded, while it is 79.3% when “uncertain” is considered an error.

## Discussion

ai systems are increasingly considered for supporting and improving the medical decision process. However, in many scenarios ai cannot (or should not) substitute the human professional^1,36^. Conversely, what is envisaged is teaming humans together with artificial intelligence to exploit the advantages of hybrid intelligence^2,3^. Would this union be able to capitalize on the respective strengths? Which are the potential factors enabling an optimal interaction? Optical diagnosis during colonoscopy represents a telling case study for answering these questions.

Endoscopists’ optical diagnosis is a psychological categorization process. Endoscopists use incoming visual information to generate possible alternative diagnoses and revise their confidence toward each. The availability of the ai advice is one more piece of information that endoscopists may actively use in this Bayesian-like revision process. We showed that endoscopists consider and are substantially influenced by ai opinion. Importantly, endoscopists can separate the good from the bad ai advice, accepting selectively more the former than the latter, as shown by a higher efficacy than safety index. This ability, combined with the relatively high accuracy of the ai classification ($\sim 85\%$), granted a beneficial effect on the overall diagnostic performance: the “hybrid human–ai teams” had, on average, better accuracy than the endoscopists alone.

How did the endoscopists select and follow the best ai advice? The successful extraction of two critical task parameters was likely at the core of this ability: endoscopists could intuitively but reliably predict for each lesion both their accuracy (not obvious^37,38^) and the accuracy of the ai (not obvious^39^). Furthermore and importantly, these prediction estimates affected endoscopists’ decisions so that they switched their diagnosis towards the ai opinion more when their confidence was low and ai perceived confidence was high. Vice-versa, endoscopists stuck with their diagnosis when their confidence was high and ai perceived confidence was low. In this way, belief revision turned out to be a sound practice in Bayesian terms, resulting in overall increased accuracy of the human–ai hybrid team.

A key to the success of hybrid teams is to calibrate human trust in ai for each specific decision. Knowing when to trust or distrust the ai allows the human expert to apply its knowledge appropriately, improving decision outcomes in cases where the ai is likely to perform poorly^4,40^. Three pitfalls undermine the beneficial effects of human–ai interaction. The first two, over-reliance or under-reliance on ai, regard a general attitude towards support systems, which is wrong when decoupled from considerations on the relative informativeness of the ai^39,41,42^. The third pitfall is more subtle and pervasive: opaque reliability of ai or human judgments, i.e., the md might not know how much s/he can trust her own, or the ai’s, judgment in each specific medical problem. If the case-by-case reliability of judgments is unknown or miscalibrated, a correct Bayesian-like belief revision could be severely hindered. Our study shows that none of these potentially dangerous patterns occurred. The endoscopists were able to build a correct mental model of the ai’s error boundaries^43^. They did so by capitalizing on explicit general warnings of ai accuracy but, more importantly, on cues in the ai output, spontaneously interpreted as informative on the ai’s confidence in its diagnosis: the persistence of the same ai diagnosis, and the rate of no-prediction ai output (Appendix B.4). md seemed to evaluate ai confidence as they would have evaluated a colleague’s by perceiving his/her hesitation^44^.

Our results could generalize to different medical settings. The given interpretation of the success of our hybrid teams stresses one enabling ingredient on the ai side, namely to provide the mds with an intuitive - yet valid - clue to ai reliability. This finding should alert decision support systems developers: ai’s perceived confidence that was unrelated to its accuracy and error boundaries^45^ might fool into error the human side of the decision team^46^. On the other hand, the absence of algorithmic transparency in the ai device considered in this study did not have a disruptive effect on md performance, arguably because mds could infer ai reliability from other indirect cues. Thus, even though algorithmic transparency has been sometimes advocated as pivotal for promoting an effective interaction with ai^6,9,36^, we suggest that easy access to case-by-case reliability may be a sufficient, or even more important, factor.

As expected, expert endoscopists showed a better performance in optical diagnosis overall standard descriptive parameters: accuracy, sensitivity, and specificity (Appendix B.2). In this context, however, the most important - and reassuring - finding was the ability to interact intuitively with ai shared between *both* expert and non-expert. The main results on influence, safety, and efficacy held for both subgroups. The benefits on accuracy were stronger for non-experts (also given their lower baseline), making their performance with ai assistance similar to that of experts without assistance. These observations also open up the interesting possibility of using ai systems for juniors’ training.

In high-stakes scenarios, such as in the medical domain, full automation of decision-making is often impossible or undesirable. This is not only for ethical or regulatory issues but also because human experts can rely on their domain knowledge, complementary to the ai’s. In hybrid decision making, the individual strengths of the human and the ai come together to optimize the joint decision outcome. In the present case study, the use of ai proved effective and safe. Effective, because mds adhered to ai’s opinions mostly when the latter were correct. Safe, because md’s adherence to ai opinions was relatively low when the latter were incorrect. When these enabling conditions are met, hybrid decision-making is an effective and appropriate diagnostic approach.

From these conclusions, we can distillate two leading suggestions. To physicians: treat ai opinion as you would treat advice from a human colleague with a slightly different expertise profile: weigh advice based on the relative historical performance between you and the colleague (i.e., how good ai has proven to be in general compared to you), but also on the colleague confidence/hesitation on the specific case. To device manufacturers: make the case-by-case confidence of the device output intuitively readable to the user.

## Supplementary Information

Supplementary Information.

Supplementary Video 1.

Supplementary Video 2.

Supplementary Video 3.

## Data Availability

To improve transparency and reproducibility of our work, raw data with de-identified participants are publicly available at OSF https://osf.io/57smj.
