# Supplementary material for: Experimental evidence of effective human–AI collaboration in medical decision-making
Source: Sci Rep. 2022 Sep 2;12:14952. doi: 10.1038/s41598-022-18751-2 (PMC9440124; doi:10.1038/s41598-022-18751-2)
Supplement: Supplementary file 1 — Supplementary Information. [file 41598_2022_18751_MOESM1_ESM.pdf]

APPENDIX
Successful human-AI integration in optical diagnosis supports hybrid intelligence for medical decision-making

Carlo Reverberi[1,2,*], **Tommaso Rigon** [3], **Aldo Solari** [2,3], **Cesare Hassan** [4,5], **Paolo Cherubini** [1,2], **GI Genius CADx Study Group**[**], **and Andrea Cherubini**[6,2,*]

[1]Department of Psychology, University of Milano–Bicocca, Milano, 20126, Italy, carlo.reverberi@unimib.it
[2]Milan Center for Neuroscience, University of Milano–Bicocca, Milano, 20126, Italy
[3]Department of Economics, Management and Statistics, University of Milano–Bicocca, Milano, 20126, Italy
[4]Digestive Endoscopy, Nuovo Regina Margherita Hospital, Rome, Italy
[5]Endoscopy Unit, Humanitas Clinical and Research Center IRCCS, Rozzano, Italy
[6]Artificial Intelligence Group, Cosmo AI/Linkverse, Lainate, 20045, Milano, Italy, acherubini@cosmopharma.com
[*]Corresponding authors
[**]A list of authors and their affiliations appears at the end of the paper

ABSTRACT

Artificial Intelligence (AI) systems are precious support for decision-making, with many applications also in the medical domain. The interaction between MDs and AI enjoys a renewed interest following the increased possibilities of deep learning devices. However, we still have limited evidence-based knowledge of the context, design, and psychological mechanisms that craft an optimal human-AI collaboration. In this multicentric study, twenty-one endoscopists reviewed 504 videos of lesions prospectively acquired from real colonoscopies. They were asked to provide an optical diagnosis with and without the assistance of an AI support system. Endoscopists were influenced by AI ($OR = 3.05$), but not erratically: they followed the AI advice more when it was correct ($OR = 3.48$) than incorrect ($OR = 1.85$). Endoscopists achieved this outcome through a weighted integration of their and the AI opinions, considering the case-by-case estimations of the two reliabilities. This Bayesian-like rational behavior allowed the human-AI hybrid team to outperform both agents taken alone. We discuss the features of the human-AI interaction that determined this favorable outcome.

A Methods

A.1 Experimental stimuli

Lesions used in the present study were acquired in a clinical study named CHANGE (Characterization Helping the Assessment of Colorectal Neoplasia in Gastrointestinal Endoscopy, Clinicaltrials.gov registration NCT04884581). CHANGE study is a single-center, single-arm, prospective study investigating the use of GI Genius CADx device in the real-time characterization of colorectal polyps (i.e., prediction of their histology during the colonoscopy). Patients enrolled underwent a standard white-light colonoscopy with the support of GIG CADx device v3.0 (manufactured by Cosmo AI/Linkverse and distributed by Medtronic). All the colonoscopy videos considered in the study were acquired in full length with unaltered quality, bearing no trace of the AI used (no overlay). Patients' clinical data and polyp histopathological information were saved in an electronic Case Report Form. The localization of each polyp in each patient was carefully annotated by scientific annotation experts. This was confronted with data in the Case Report Form for the same patient to avoid any possibility of erroneous correspondence between polyp in the video and the related histology. Polyps for which video recording failed or for which no histology could be obtained were excluded. For each polyp, a short video clip was prepared, starting a few seconds before the first polyp appearance and ending with endoscopic polyp resection. If multiple polyps were present in the same video section, a separate clip was generated for each individual polyp. For each lesion, two different video clip versions were prepared. In a first version, to be used in `S1`, the clip had no markings except for a green box surrounding the target lesion for the first ten seconds after the lesion appearance. In the second version, to be used in `S2`, the video clip was processed with GIG algorithms, and the output was identical to that observed during real-life utilization of the GIG medical device. The labels analyzing/adenoma/non-adenoma/no-prediction were visible on the lesion.

A.1.1 Example video clips

We provide in the supplementary online materials three video clips from the experimental stimuli set. The output of the AI visible in the video clips is the original generated by the CADx, and it was shown in Session S2. The same lesion was previously shown in Session S1 without the label of the CADx, i.e., only with the CADe green box.

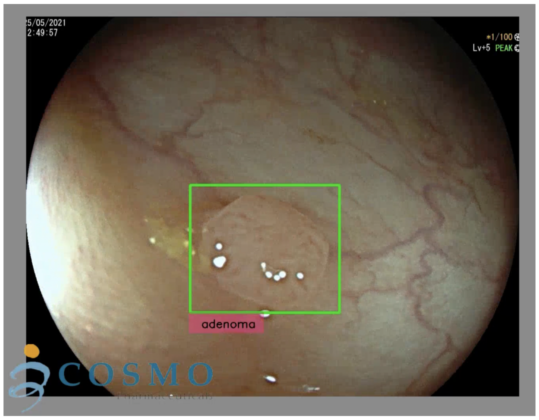

Figure A.1. Frame of a video clip of a colonoscopy showing a lesion automatically labeled by the AI system as "adenoma". The full video clip is available in the supplementary material online as *V1_example_adenoma.mp4*.

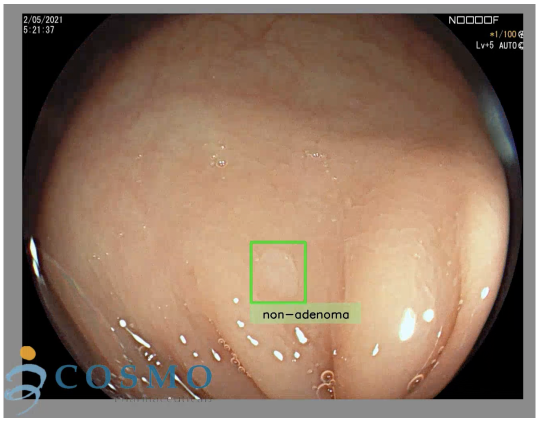

Figure A.2. Frame of a video clip of a colonoscopy showing a lesion automatically labeled by the AI system as "non-adenoma". The full video clip is available in the supplementary material online as *V2_example_nonadenoma.mp4*.

A.1.2 GI Genius CADx device

The CADx+CADe device used in this study is GI Genius v3.0 (developed by Cosmo AI, Ireland, and distributed by Medtronic, US). GI Genius v3.0 received CE clearance under the European Medical Device Directive (MDD, 93/42/EEC) in 2021 as a class IIa medical device. The CADx is designed to automatically activate when a new polyp is detected by a CADe algorithm in a colonoscopy video stream. For each polyp, a CADx algorithm overlays a frame-by-frame live decision specifying its binary histology ("adenoma" or "non-adenoma"). The CADx can also abstain from predicting the polyp histology in a frame either by printing "no-prediction" if the system is unsure about the histology or "analyzing" if an insufficient number of features across multiple frames was detected. The CADx algorithm training details and its performances are fully reported by the manufacturer (Linkverse, Cosmo AI) in the documentation accompanying the device. Briefly, the CADx neural network was trained using data extracted from the study "The Safety and Efficacy of Methylene Blue MMX Modified Release Tablets Administered to Subjects Undergoing Screening or Surveillance Colonoscopy" (ClinicalTrials.gov NCT01694966), a multinational, multicenter study that enrolled over one thousand patients. The study recorded lossless, high definition, full procedure colonoscopy videos and complete information on polyp characteristics and histology. The histopathological evaluation was based on the revised Vienna classification of gastrointestinal epithelial neoplasia. The polyps corresponding to Vienna category 1 (negative for neoplasia) or 2 (indefinite for neoplasia) were considered "non-adenoma". The polyps corresponding to Vienna category 3

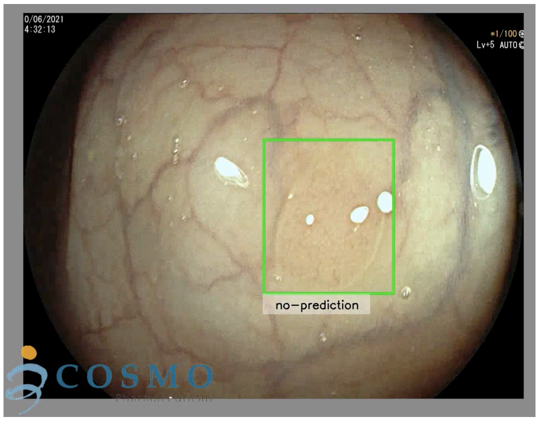

Figure A.3. Frame of a video clip of a colonoscopy showing a lesion automatically labeled by the AI system as "no-prediction". The full video clip is available in the supplementary material online as *V3_example_noprediction.mp4*.

(low-grade mucosal neoplasia), 4 (high-grade mucosal neoplasia), or 5 (submucosal invasion of neoplasia), were considered "adenoma". The video dataset thus obtained was further split into training, validation, and test subsets. Frames containing a polyp were manually annotated by trained personnel. Overall, for training, we collected 345 patients, 957 polyps, and 63445 images; for validation, we had 44 patients, 133 polyps, and 8645 images; for test, 165 patients, 405 polyps, and 26412 images.

A.2 Sampling plan and data collection

A.2.1 Overview

After protocol registration, we sent invitations to expert and non-expert reviewers. Experts were endoscopists having more than 5 years of colonoscopy experience, a proven track of scientific publications on optical characterization or a similar subject, and experience in optical biopsy with virtual chromoendoscopy. An endoscopist was considered "non-expert" if s/he had performed less than 500 colonoscopies. We sent following waves of 20 invitations until we surpassed the minimum target number of participants (16 participants, 8 experts, and 8 non-experts). We sent 40 invitations, 25 endoscopists accepted the invitation, and 21 completed the review of all lesions within the deadline. The average time needed to complete the survey was 55 days.

A.2.2 Procedure

Each batch of 84 lesions (`B01`, `B02`, ..., `B06`) was administered as `S1` and as `S2`. At least two weeks passed from the conclusion of the evaluation of one batch in `S1` and the evaluation of the same batch in `S2`. The sequence of administration was the following:

```
B01_S1, B02_S1, B03_S1,
B01_S2, B02_S2, B03_S2,
B04_S1, B05_S1, B06_S1,
B04_S2, B05_S2, B06_S2.
```

For each batch, 21 pre-randomization lists were prepared for the order of presentation of lesions. Each reviewer was preassigned a pre-randomization `ID`, and lesions in each batch were presented to the reviewer with the order corresponding to the pre-randomization `ID`.

A.2.3 Questions of the online survey

Data were collected through an online survey. Each participant was provided with a unique username. The video interface allowed the user to fully control what to play, allowing the endoscopist to skip, view again, and pause. We recorded the viewing time for each frame. The participants answered the following questions.

```
- SESSION 1

A. Please classify the lesion in the video in one of the following categories:
1. Adenoma
2. Hyperplastic
3. SSL
4. Carcinoma
5. Uncertain

B. Your level of confidence relative to question A:
1. Very high confidence
2. High confidence
3. Low confidence
4. Very low confidence
```

```
- SESSION 2

A. Please classify the lesion in the video in one of the following categories:
1. Adenoma
2. Hyperplastic
3. SSL
4. Carcinoma
5. Uncertain

B. Your level of confidence relative to question A:
1. Very high confidence
2. High confidence
3. Low confidence
4. Very low confidence

C. Please report what the output of the AI about the lesion was:
1. AI's output was  "Adenoma"
2. AI's output was "Non-Adenoma"
3. AI output was "Uncertain"
4. I am not sure / I did not notice the AI output

D. What do you think was the level of confidence in the AI output? The AI output showed:
1. very high confidence
2. high confidence
3. low confidence
4. very low confidence
```

Question B was offered only in case the participant did not choose 5 in question A. Question D was offered only in case the participant did not choose 4 in question C. All response times were logged. It was not possible to change the answer to one question once it had been recorded.

A.2.4 Timing

We started sending invitations to reviewers on June 24, 2021. We started analyzing data only when the entire data collection was completed in October 2021. Before completing data collection, an employee of Cosmo AI/Linkverse - not involved in writing the paper or analyzing data - monitored data collection for completeness and potential errors.

A.3 Formal definition of the transformed variables

Let $J = 21$ be the number of subjects (endoscopists) considered in the study, and let $n = 504$ be the number of lesions that will be shown to each subject. For each of the J subjects and n lesions, the values of the *histologic evaluation* variable (Tab. 1) are denoted with T_i, for $i = 1,\dots,n$. Moreover, the values of the *human judgment, session 1 and session 2* variables (Tab. 1) are denoted with H_{ij1}, H_{ij2}, respectively, for $i = 1,\dots,n$ and $j = 1,\dots,J$. Finally, the values of the *perception of* AI *prediction* variable (Tab. 1) are denoted with A_{ij} for $i = 1,\dots,n$ and $j = 1,\dots,J$.

We say that the jth subject correctly identified the presence / absence of an adenoma in the ith lesion in `S1` whenever $H_{ij1} = T_i$ and whenever $H_{ij2} = T_i$ at `S2`. Similarly, we say that the AI provided the correct diagnosis according to the jth subject and for the ith lesion whenever $A_{ij} = T_i$. In this setting, note that we were assuming that the "Uncertain" and "Undetermined" outcomes were regarded as wrong diagnoses. Let $\mathbb{1}(\cdot)$ be the indicator function. We created the following transformed binary variables, that are also described in Tab. 1. The binary variable $D_{ijk} \in \{0,1\}$ is defined as

$$D_{ijk} = \mathbb{1}(H_{ij1} = T_i), \qquad i = 1,\dots,n, \quad j = 1,\dots,J, \quad k = 1,2,$$

and indicates whether the ith lesion is correctly diagnosed ($D_{ijk} = 1$) or not ($D_{ijk} = 0$) by the jth subject (endoscopist) at the kth session. We call this variable *human correct diagnosis, session 1 and session 2*. Secondly, we let the binary variable $D_{ij,\mathrm{AI}} \in \{0,1\}$ be

$$D_{ij,\mathrm{AI}} = \mathbb{1}(A_{ij} = T_i), \qquad i = 1,\dots,n,$$

indicating whether the ith lesion is correctly diagnosed ($D_{ij,\text{AI}} = 1$) or not ($D_{ij,\text{AI}} = 0$) by the AI. This quantity is referre to as AI *(perceived) correct diagnosis*. Finally, the discrete numerical variable $S_{ijk} \in \{1, \ldots, 9\}$ measures the belief of the jth endoscopist about the ith lesion during the kth session. For example, the score $S_{ijk} = 9$ indicates a strong belief of the jth endoscopist in session k that the ith lesion is an adenoma. Viceversa, $S_{ijk} = 1$ denotes a strong belief of the jth endoscopist in session k that the ith lesion is not an adenoma. The confidence score is defined as a combination of the *human judgment* variables and the associated *human confidence* variables. Hence, we let $S_{ijk} = 1, 2, 3, 4$ when the associated judgement is "Non-Adenoma" and the confidence ranges between "Very high" to "Very low". Similarly, we let $S_{ijk} = 6, 7, 8, 9$ when the associated judgement is "Adenoma" and the confidence ranges between "Very low" to "Very high". We set $S_{ijk} = 5$ when the judgments and hence the confidence are "Uncertain". We call these quantities *confidence scores* for `S1` and `S2`.

A.4 Statistical modeling of the main hypotheses

A.4.1 Definition of the primary inferential quantities

We were interested in comparing the probability of a certain event during session 1 with the probability of the same event during session 2, possibly considering a subset of the observations. We made use of odds ratios and we define the function $\text{odd}(\pi) = \pi/(1-\pi)$ for any $\pi \in (0, 1)$. For example, the main hypothesis (2.) in Section 2.3 aims at comparing the probabilities of the *correct diagnosis* between `S1` and session 2. We define the odds ratio

$$(\text{"AI effect on accuracy"}) = \frac{\text{odd}\{\text{pr}(D_{ij2} = 1)\}}{\text{odd}\{\text{pr}(D_{ij1} = 1)\}}, \tag{1}$$

for $j = 1, \ldots, J$ and $i = 1, \ldots, n$. This odds ratio captures the magnitude of AI's effect on diagnosis. If its value is greater than (less than) 1 it means that AI's assistance is associated with an increased (decreased) probability of a correct diagnosis. Hence, this quantity is the main measure of interests for addressing the main hypothesis (2.) described in Section 2.3.

The second important aspect of the analysis is the estimation and quantification of the *effectiveness* and *safety* measures, which represent the AI *effect on the diagnostic accuracy* conditionally on the fact that the AI was correct (effectiveness) or incorrect (safety). These indices corresponds to the main hypothesis (3.) and (4.) in Section 2.3. We defined the *effectiveness* as the following odds ratio

$$(\text{"Effectiveness"}) = \frac{\text{odd}\{\text{pr}(D_{ij2} = 1 \mid D_{ij,\text{AI}} = 1)\}}{\text{odd}\{\text{pr}(D_{ij1} = 1 \mid D_{ij,\text{AI}} = 1)\}}, \tag{2}$$

for any $i \in \mathscr{E}_j$ and $j = 1, \ldots, J$, where we define the index set $\mathscr{E}_j = \{i = 1, \ldots, n : D_{ij,\text{AI}} = 1\}$. Similarly for the *safety* we let

$$(\text{"Safety"}) = \frac{\text{odd}\{\text{pr}(D_{ij2} = 1 \mid D_{ij,\text{AI}} = 0)\}}{\text{odd}\{\text{pr}(D_{ij1} = 1 \mid D_{ij,\text{AI}} = 0)\}}, \tag{3}$$

for any $i \in \mathscr{S}_j$ and for $j = 1, \ldots, J$, where we define the index set $\mathscr{S}_j = \{i = 1, \ldots, n : D_{ij,\text{AI}} = 0\}$. The last important aspect of our analysis was the *influence* of the AI, irrespective of its correctness, as described in hypothesis (1.) in Section 2.3. Once again we relied on odds ratios and we defined:

$$(\text{"AI influence"}) = \frac{\text{odd}\{\text{pr}(H_{ij2} = A_{ij})\}}{\text{odd}\{\text{pr}(H_{ij1} = A_{ij})\}}, \tag{4}$$

for $i \in \mathscr{A}_j = \{1, \ldots, n : A_{ij} \notin \{\text{"Uncertain", "I did not notice"}\}\}$ and for $j = 1, \ldots, J$, representing a measure of the shift of opinion of the endoscopist towards the perceived AI response.

The measures presented in this section (AI effect on accuracy, *effectiveness*, *safety*, AI *influence*) do not depend neither on the endoscopist nor on the lesions. This important assumption is a consequence of the logistic regression specification discussed in A.4.2 and crucially facilitates the interpretation of these indices.

A.4.2 Logistic regression modeling

We present the statistical modeling in an abstract fashion, as all the main hypothesis described in Section 2.3 (*influence*, *diagnostic accuracy*, *effectiveness and safety*), can be tested by relying on the same statistical tools. The quantities described in the previous section involve independent binary indicators $y_{ijk} \in \{0, 1\}$, associated to the ith lesion, the jth endoscopist, and the kth session. Moreover, recall that we are interested in estimating odds ratios of the form $\text{odd}\{\text{pr}(y_{ij2} = 1)\}/\text{odd}\{\text{pr}(y_{ij1} = 1)\}$. Let us define the following probabilities

$$\pi_{ijk} = \text{pr}(y_{ijk} = 1), \qquad i \in \mathscr{I}_j, \qquad j = 1, \ldots, J, \qquad k = 1, 2, \tag{5}$$

where we consider a subject-specific generic subset $\mathscr{I}_j \subseteq \{1,\ldots,n\}$. Then, we assume the following logistic regression specification

$$\log\left(\frac{\pi_{ij1}}{1-\pi_{ij1}}\right) = \mu_i + \alpha_j, \qquad \log\left(\frac{\pi_{ij2}}{1-\pi_{ij2}}\right) = \mu_i + \alpha_j + \beta, \qquad i \in \mathscr{I}_j,$$

and for $j = 1,\ldots,J$. We regarded β as an unknown parameter, whereas the lesion-specific μ_i and subject-specific α_j values were modelled as Gaussian random effects for $i \in \mathscr{I}_j$ and $j = 1,\ldots,J$. This specification leads to a straightforward interpretation β in terms of odds ratio. In fact, we can express the key parameter as follows

$$(\text{“AI influence”}) = \frac{\text{odd}\{\text{pr}(y_{ij2}=1)\}}{\text{odd}\{\text{pr}(y_{ij1}=1)\}} = \omega = e^{\beta}.$$

for any $i \in \mathscr{I}_j$ and $j = 1,\ldots,J$. We use the following notation for these key parameters:

$$(\text{“AI influence”}) = \omega_I, \tag{6}$$
$$(\text{“AI effect on accuracy”}) = \omega_A, \tag{7}$$
$$(\text{“Effectiveness”}) = \omega_E, \tag{8}$$
$$(\text{“Safety”}) = \omega_S. \tag{9}$$

A.4.3 Estimation and testing procedure

The models we described are logistic regression models with (Gaussian) random effects. The estimated parameters were obtained via (integrated) maximum likelihood and exploiting the `lme4` R package[1]. Related inferential quantities (e.g. confidence intervals, hypothesis testing) were computed using the `lme4` and/or `exact2x2` R packages[2].

A.4.4 Inferential goals

The primary inferential goals were the main hypotheses (1.), (2.), (3.), and (4.) discussed in Section 2.3:

1. *Influence of the* AI. The opinion of the endoscopist is influenced by the response of the AI. This amounts to testing the following hypothesis $H_0 : \omega_I \leq 1$ against $H_1 : \omega_I > 1$, where ω_I represents the AI *influence* as defined in equation (6).

2. *Diagnostic accuracy*. If the interaction between endoscopists and AI is well-calibrated, the diagnostic accuracy should improve. This amounts to testing the null hypothesis $H_0 : \omega_A \leq 1$ against the alternative $H_1 : \omega_A > 1$, where ω_A represents the AI *effect on the diagnostic accuracy* defined in equation (7).

3. *Effectiveness*. The endoscopists are rightfully accepting the AI opinion when this is correct,. This amounts to testing the null hypothesis $H_0 : \omega_E \leq 1$ against the alternative $H_1 : \omega_E > 1$, where ω_E represents the *effectiveness* defined in equation (8).

4. *Safety*. The endoscopists are able to disengage from a wrong opinion of the AI. This amounts to testing the null hypothesis $H_0 : \omega_S \leq \Delta_S$ against $H_1 : \omega_S > \Delta_S$, where ω_S represents the safety defined in equation (9) and Δ_S is the safety limit. We let $\Delta_S = 0.3$.

The aforementioned null hypotheses were tested sequentially at level α with a fixed-sequence multiple testing procedure[3]. If a certain hypothesis is rejected at the α level, the next hypothesis in the sequence is tested at the same level. Otherwise, testing stops, and subsequent hypotheses are not tested. The fixed-sequence procedure controls the family-wise error rate at level α; namely, the probability of at least one incorrect conclusion (type I error) is bounded by α. For the inference on a generic odds ratio ω, we calculated the p-value for $H_0 : \omega \leq 1$ ($H_0 : \omega \leq \Delta$) and the point estimate $\hat{\omega}$ along with the $(1-\alpha)\%$ confidence lower bound $\underline{\omega}_\alpha$. If we observe a p-value less than α and/or $\underline{\omega}_\alpha > 1$ ($\underline{\omega}_\alpha > \Delta$), then $H_0 : \omega \leq 1$ ($H_0 : \omega \leq \Delta$) will be rejected at level α. The significance level α is set to 5%.

A.5 Power analysis for main inferential goals

The study is successful when there is a positive outcome in all endpoints: AI influence, AI effect on accuracy, effectiveness, and safety. In the following power analysis, we will focus on the safety endpoint. Given the fewer observations available, the safety endpoint is the least powered analysis of all endpoints. Thus, it provides a lower bound for the power of the other endpoints.

We performed a simulation-based power analysis for the safety endpoint based on the logistic regression model described in Section A.4.2. We considered (i) a prospective power calculation based on parameter estimates derived from a pilot study and (ii) a retrospective power calculation based on parameter estimates derived from the current study.

	J	n_S	$\omega_S = e^{\beta}$	μ	σ^2_{μ}	σ^2_{α}
Prospective power analysis (pilot study)	16	100	0.686	0.792	2.824	0.098
Retrospective power analysis (main study)	21	100	0.542	1.310	2.502	0.115

Table A.1. Parameter values used in the simulation-based power analysis for the safety endpoint: number of subjects (endoscopists) J, number of lesions relevant to safety n_S, safety effect size $\omega_S = e^{\beta}$, intercept term μ and variances σ^2_{μ} and σ^2_{α} of the lesion and the subject random-effects.

The parameters required for the power analysis are the number of subjects (endoscopists) J, the number of lesions relevant to safety n_S (i.e., the number of AI incorrect diagnoses), safety effect size $\omega_S = e^{\beta}$, the intercept term μ and the variances σ^2_{μ} and σ^2_{α} of the lesion and the subject random-effects.

Table A.1 shows the parameter values estimated from the pilot study (prospective power analysis) and the main study (retrospective power analysis). The pilot study involved $J = 5$ subjects (endoscopists) evaluating $n = 509$ lesions, where the number of lesions relevant to the safety endpoint is $n_S = 147$. However, in the prospective power calculation, we used $n_S = 100$ since the main study would use the next version of the AI engine, featuring a higher AI accuracy. The estimate for β from the pilot study is -0.1205 with a standard error of 0.128; we used the β estimate minus twice the standard error for the safety effect size. The number of observations obtained by the subject-lesion combination is $16 \times 100 = 1600$ for the prospective analysis and $21 \times 100 = 2100$ for the retrospective analysis. The parameters specification for the retrospective analysis gives a probability of correct diagnosis in the subset of lesions where AI is wrong equal to 78.8% in `S1` and 66.8% in `S2`, on average over lesions and subjects. The parameters specification for the prospective analysis gives a probability of correct diagnosis in the subset of lesions where AI is wrong equal to 68.8% in `S1` and 60.2% in `S2`, on average over lesions and subjects. The safety effect size estimate of 0.686 based on the pilot study is higher than the estimate of 0.542 based on the main study, and this may be partly due to the lower probability of a correct diagnosis in the subset of lesions where AI is wrong in the pilot study.

The safety endpoint is achieved when the lower bound $\underline{\omega}_S$ of the 95% confidence interval for the safety effect size ω_S is greater than the safety limit Δ_S. We declared in the preregistration a safety limit $\Delta_S = .3$. Fig. A.4 displays the probability of success for the safety endpoint (power) as a function of the safety limit Δ_S. Power is estimated by simulation, using 1000 replications, by setting the parameter values in Tab. A.1. For the declared safety limit $\Delta_S = .3$, the estimated power is $> 99\%$ both in the prospective and the retrospective analyses. In conclusion, a sample size $J = 16$ warrants adequate power for our main endpoints.

A.6 Statistical modeling of ancillary effects on endoscopist beliefs

A further refinement of the change in agreement is possible by conditioning on the outcomes of the AI, i.e. when AI $= d$ with $d \in \{$ "Adenoma", "Non-Adenoma"$\}$. We define

$$(\text{“Change in agreement when AI} = d\text{”}) = \frac{\text{odd}\{\text{pr}(H_{ij2} = A_{ij} \mid A_{ij} = d)\}}{\text{odd}\{\text{pr}(H_{ij1} = A_{ij} \mid A_{ij} = d)\}}, \tag{10}$$

for any $i \in \mathscr{D}_j = \{1, \dots, n : A_{ij} = d\}$ and for $j = 1, \dots, J$. The odds ratio represents the shift of opinion towards the perceived AI response when AI $= d$. We adopted the logistic model specification described in A.4.2 by conditioning on AI $= d$, for $d \in \{$"Adenoma", "Non-Adenoma"$\}$.

We were also interested in comparing the measure of belief of the jth endoscopist about the ith lesion in session 1, encoded in the confidence score S_{ij1}, with the measure of belief expressed in session 2, encoded in the confidence score S_{ij2}. For estimating the average AI effect on the confidence score when AI $= d$ with $d \in \{$"Adenoma", "Non-Adenoma"$\}$, we defined

$$(\text{“Effect on confidence score when AI} = d\text{”}) = \mathbb{E}(S_{ij2} \mid A_{ij} = d) - \mathbb{E}(S_{ij1} \mid A_{ij} = d), \tag{11}$$

for any $i \in \mathscr{D}_j = \{1, \dots, n : A_{ij} = d\}$ and for $j = 1, \dots, J$. Then, we assumed the following linear regression specification

$$\mathbb{E}(S_{ij1} \mid A_{ij} = d) = \tau_i + \kappa_j, \qquad \mathbb{E}(S_{ij2} \mid A_{ij} = d) = \tau_i + \kappa_j + \delta, \tag{12}$$

where τ_i, κ_j are nuisance parameters that will be modelled as Gaussian random effects and fixed effects, respectively. The key parameter δ captures the shift in confidence score between the two sessions when AI $= d$, with δ greater than (less than) 0 indicates that AI $= d$ is associated with an increased (decreased) confidence towards "Adenoma".

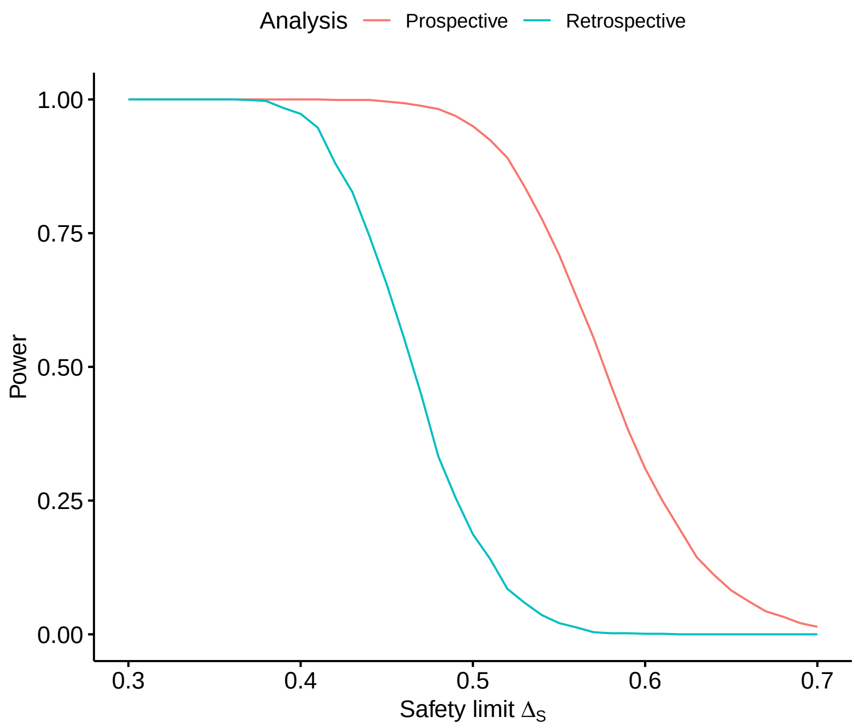

Figure A.4. Power for the safety endpoint as a function of the safety limit Δ_S for the prospective and retrospective analyses.

B Results

B.1 Endoscopists' processing performance

We asked whether the time required to judge a lesion is impacted by the availability of AI opinions. Endoscopists took, on average, a similar amount of time to decide in S1 and in S2. Notwithstanding that the presence of AI has a minimal effect on decision times, these are modified by other factors. Namely, confidence and the agreement between endoscopists and AI. Endoscopists take longer to decide, in both sessions, when they are less confident (Fig. B.5), and they take longer when their opinions disagree with AI (Fig. B.6). These effects on decision times may be understood by considering the relative difficulty of the lesions. On the one hand, when lesion difficulty is taken into account, the effect of confidence on decision times mostly disappears. On the other, the "disagreement effect" in S2 almost disappears when it is assessed by discarding the decision times of the same lesion subgroups in S1 (when no disagreement with AI is possible). This suggests that a large part of the "disagreement effect" was due to the differential lesion difficulty in the agreement and disagreement subgroups.

Non-experts were faster than experts both in S1, and in S2 (Fig. B.7). However, experts' responses slowed down in S2 more than non-experts' responses, meaning that experts spent more time in assessing AI's opinions.

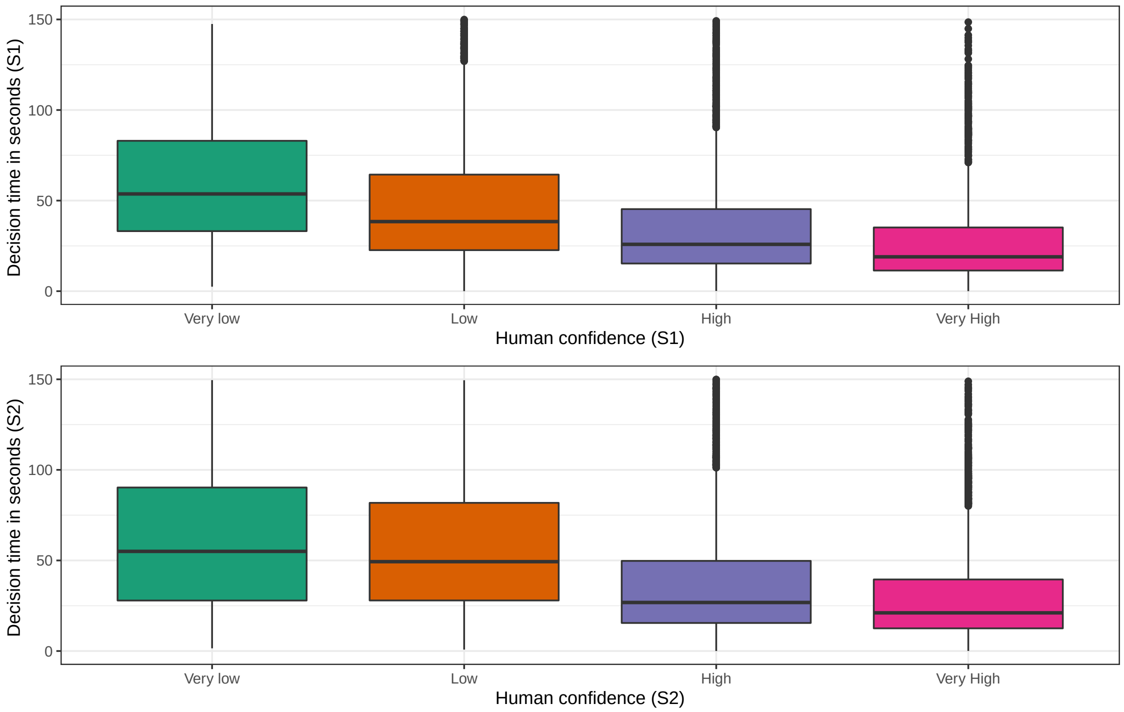

Figure B.5. Decision times expressed in seconds (excluding values > 150 seconds), in both Sessions S1 and S2 and for different confidence levels.

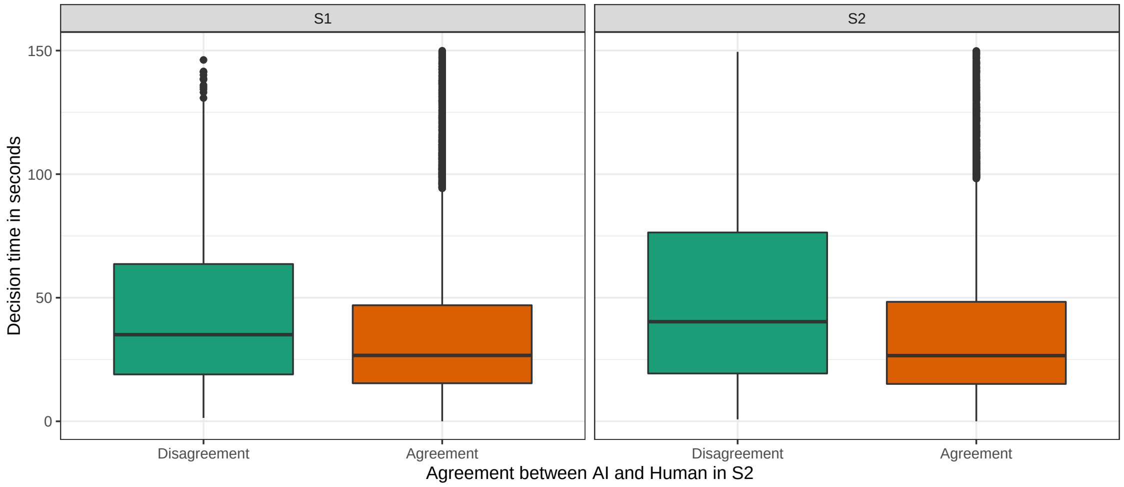

Figure B.6. Decision times expressed in seconds (excluding values > 150 seconds), in both Sessions S1 and S2 and grouped for agreement between AI and the endoscopist in S2.

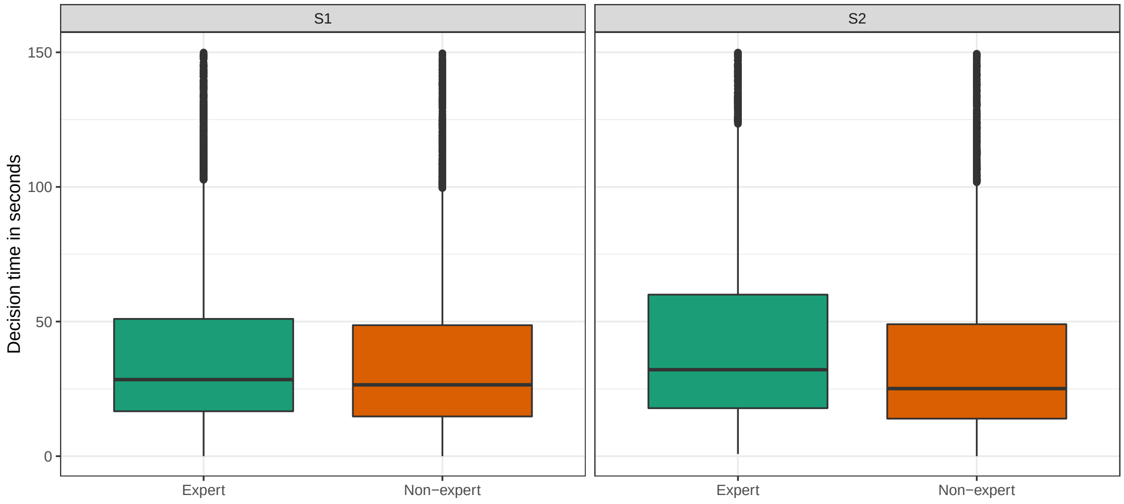

Figure B.7. Decision times expressed in seconds (excluding values > 150 seconds), in both Sessions `S1` and `S2` and grouped for expertise.

Endoscopist	Expertise	ω_I	ω_A	ω_E	ω_S
A	expert	*101.00*	1.72	∞	*0.24*
B	expert	1.73	1.12	1.60	0.80
C	non-expert	*6.25*	1.88	*8.00*	*0.30*
D	non-expert	*5.33*	1.63	*4.60*	1.07
E	non-expert	*2.67*	1.51	*2.53*	0.88
F	non-expert	*3.90*	1.70	*3.50*	0.65
G	expert	*3.69*	1.29	*3.80*	0.48
H	expert	*6.60*	1.55	*12.00*	0.50
I	expert	1.78	0.83	1.75	*0.27*
J	expert	*2.70*	1.19	2.43	0.57
K	non-expert	*115.00*	*3.96*	∞	*0.17*
L	expert	*7.00*	0.86	*7.67*	*0.35*
M	non-expert	*1.83*	1.47	2.06	0.80
N	non-expert	*3.69*	1.09	*3.50*	0.57
O	non-expert	*3.58*	0.98	*3.44*	*0.28*
P	non-expert	*3.48*	1.77	*3.41*	0.50
Q	expert	*4.44*	1.38	*4.27*	*0.35*
R	non-expert	*10.20*	*3.36*	*12.00*	0.48
S	expert	*6.88*	0.84	*3.86*	*0.13*
T	non-expert	*3.38*	1.91	*4.25*	0.57
U	expert	*9.25*	1.20	*10.00*	0.22

Table B.2. Estimated odds-ratio ω_I (influence), ω_A (accuracy), ω_E (effectiveness) and ω_S (safety) for individual endoscopists. Odds-ratio estimates in italics are significantly different from 1 at level 5% after Hommel's multiplicity correction across the 21 subjects (i.e. adjusted p-value < 0.05).

B.2 Individual variability

This section explores whether the behavioral patterns observed in our global analyses also hold for each endoscopist.

Estimated odds-ratio ω_I (influence), ω_A (accuracy), ω_E (effectiveness) and ω_S (safety) for individual endoscopists are reported in Tab. B.2, Fig. B.8 and Fig. B.9 Estimated marginal probability (i.e. proportions) of agreement, accuracy, effectiveness, safety and AI perceived accuracy for individual endoscopists in S1 and S2 are reported in Fig. B.10). The great majority of endoscopists increased their accuracy in S2 with respect to S1 (17 out of 21, that is about the 81%). Expert endoscopist were in general more confident than non-experts both towards themselves and toward AI (Fig. B.11). Overall, the reported individual-level observations encourage the conclusion that our main findings are robust to the effect of individual variability.

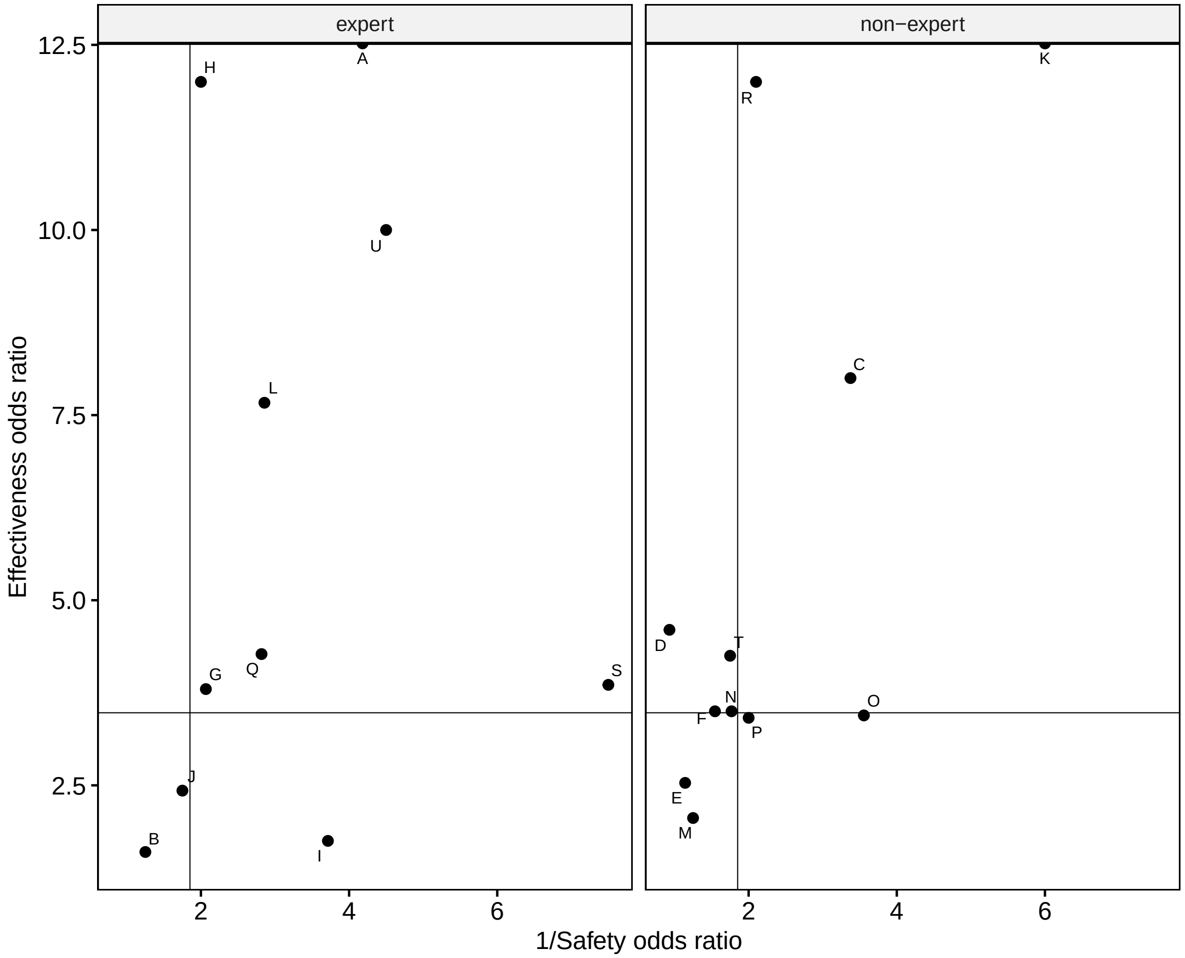

Figure B.8. Estimated odds ratio ω_E (Effectiveness) and $1/\omega_S$ (1/Safety) for individual endoscopists, labelled A, B, ..., U, separately by expertise. The axes correspond to the overall values $(1/\omega_S, \omega_E) = (1/0.54, 3.48)$.

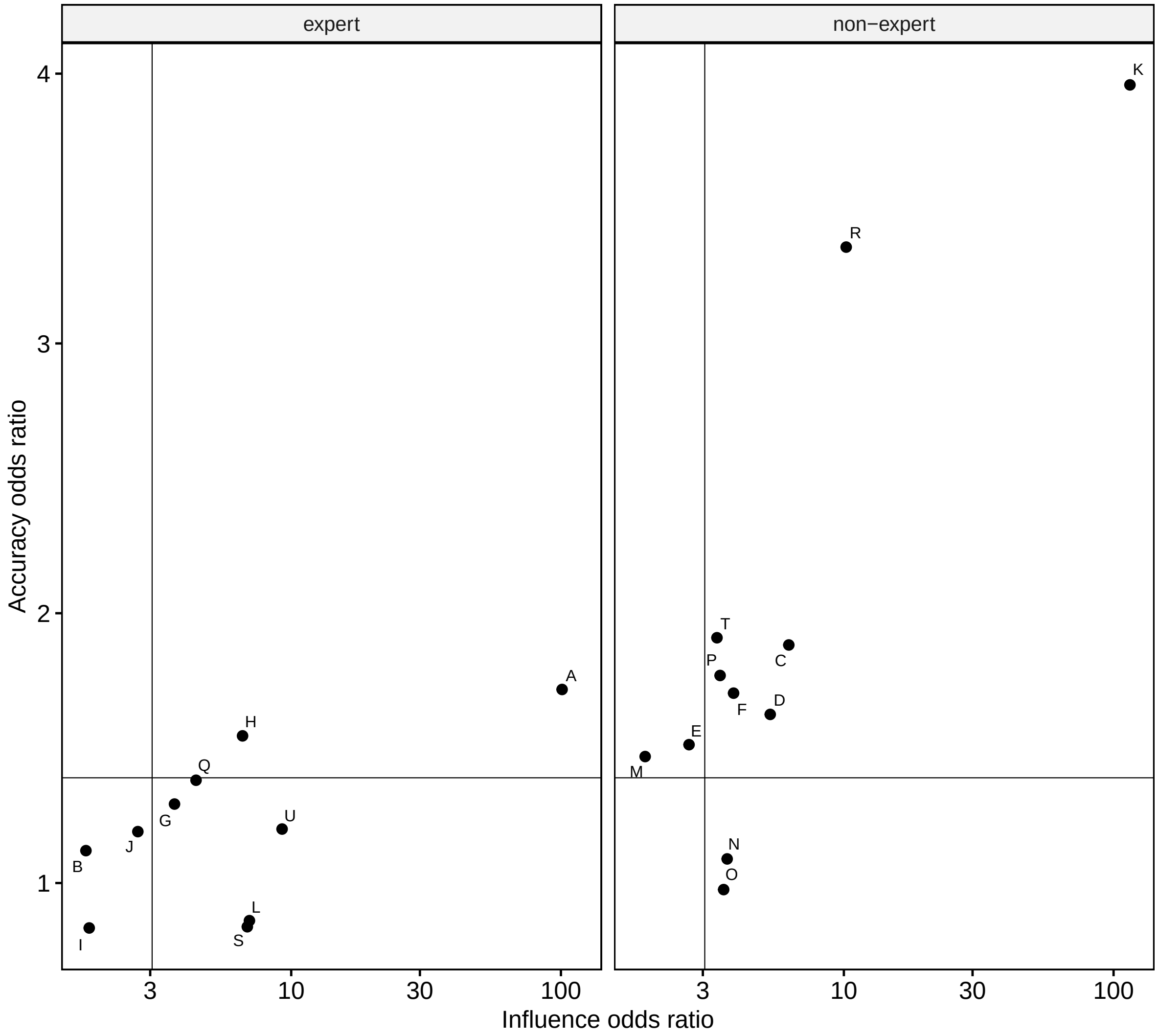

Figure B.9. Estimated odds ratio ω_I (Influence) and ω_A (Accuracy) for individual endoscopists, labelled A, B, ..., U, separately by expertise. The axes correspond to the overall values $(\omega_I, \omega_A) = (3.05, 1.39)$.

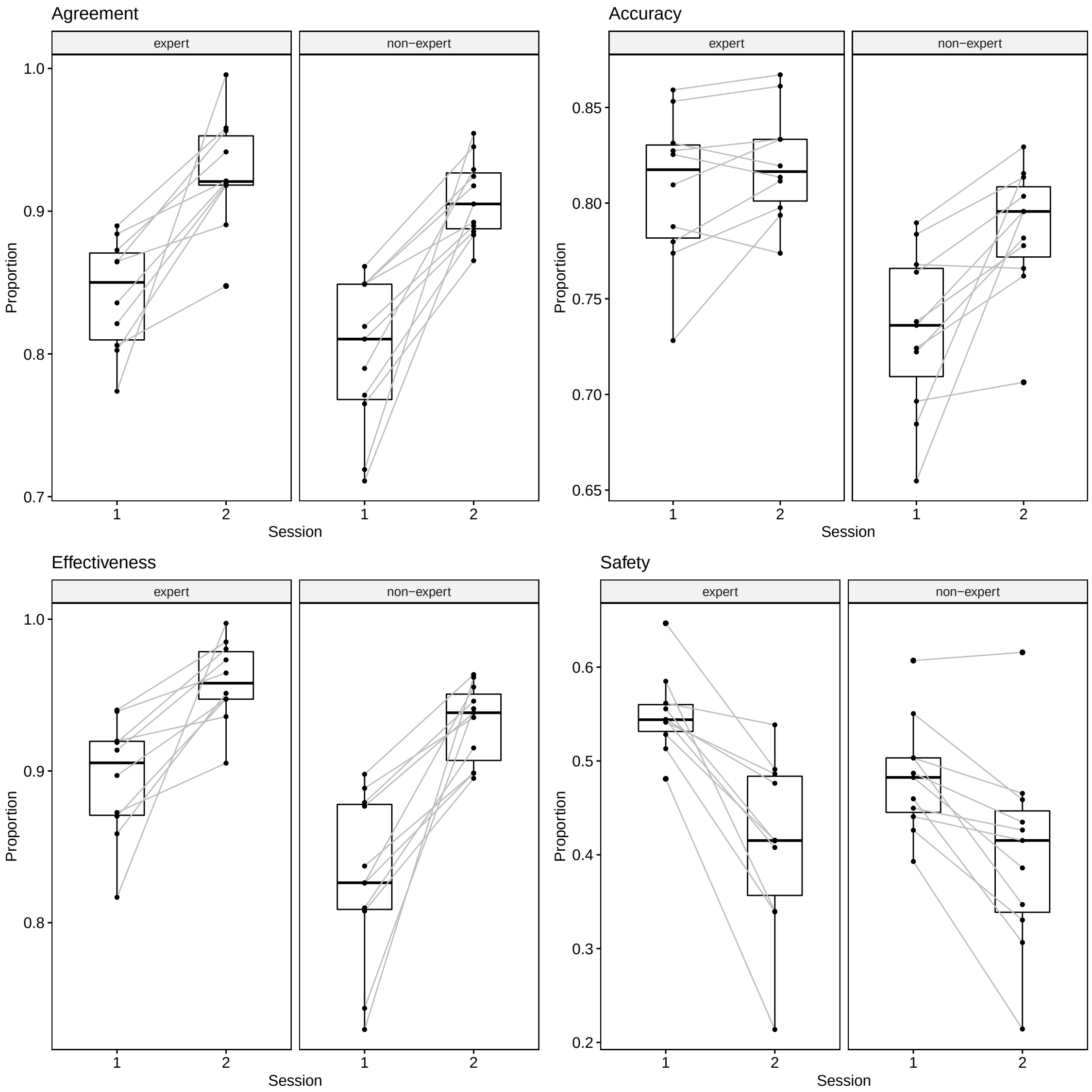

Figure B.10. Estimated marginal probability (i.e. proportions) of agreement, accuracy, effectiveness and safety in Session `S1` and `S2` for individual endoscopists, separately by expertise.

Endoscopist	Expertise	S1 accuracy	S2 accuracy	AI accuracy$_u$	AI accuracy$_s$
Code: A	expert	0.73	0.79	0.74	0.82
Code: B	expert	0.83	0.83	0.74	0.87
Code: C	non-expert	0.74	0.80	0.75	0.84
Code: D	non-expert	0.77	0.81	0.54	0.88
Code: E	non-expert	0.74	0.78	0.74	0.88
Code: F	non-expert	0.72	0.76	0.68	0.90
Code: G	expert	0.77	0.80	0.72	0.87
Code: H	expert	0.81	0.83	0.71	0.88
Code: I	expert	0.79	0.77	0.73	0.85
Code: J	expert	0.85	0.86	0.78	0.86
Code: K	non-expert	0.65	0.80	0.78	0.81
Code: L	expert	0.83	0.81	0.67	0.86
Code: M	non-expert	0.78	0.81	0.77	0.83
Code: N	non-expert	0.70	0.71	0.57	0.82
Code: O	non-expert	0.77	0.77	0.71	0.87
Code: P	non-expert	0.72	0.78	0.77	0.84
Code: Q	expert	0.78	0.81	0.77	0.82
Code: R	non-expert	0.68	0.82	0.77	0.82
Code: S	expert	0.83	0.82	0.79	0.83
Code: T	non-expert	0.79	0.83	0.77	0.86
Code: U	expert	0.86	0.87	0.80	0.84

Table B.3. Human diagnostic accuracy (in S1 and in S2) and AI accuracy of the perceived diagnosis. Following the standard in the field, AI accuracy$_s$ does not consider as wrong lesions where AI opinion was perceived as "uncertain" or it was "not noticed". AI accuracy$_u$ more conservatively reports accuracy by considering "uncertain" and "not noticed" as wrong. The difference between the two AI accuracies is thus due to the varying proportion of lesions classified as "uncertain" and "not noticed" by each endoscopist.

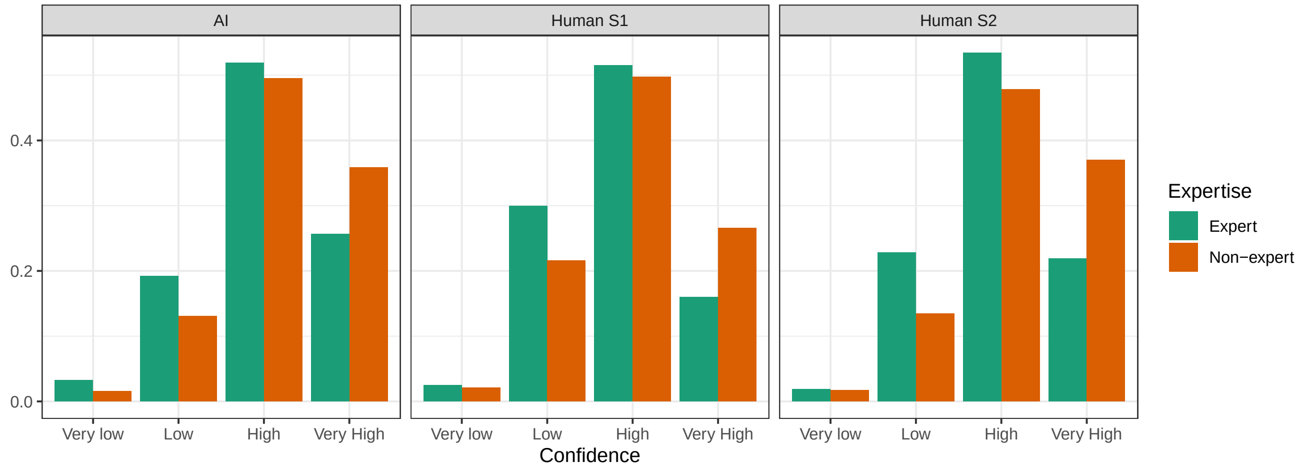

Figure B.11. Confidence distribution in expert and non-expert endoscopists towards themselves (Session S1, S2), and towards AI.

B.3 Effects on endoscopists beliefs

A well-calibrated endoscopist should always consider and integrate informative opinions of the AI. This should be visible in the final endoscopist decisions. However, even when endoscopists do not change their decision, we should observe a belief modification on a more fine-grained scale, such as the confidence scores. Namely

1. The *change in confidence - adenoma*. We expected a shift of the endoscopist "confidence score" towards adenoma (i.e. towards score 9) when AI opinion equals "Adenoma".
2. The *change in confidence - non-adenoma*. We expected a shift of the endoscopist "confidence score" towards the non-adenoma (i.e. towards score 1) when AI opinion equals "Non-Adenoma".

Results are reported in Tab. B.4. On average, the confidence score increases by 0.72 when the perceived AI response is "Adenoma", and decreases by 0.73 when "Non-Adenoma". Fig. B.12 displays confidence score frequency distribution in the two sessions S1 and S2 as a function of perceived AI response.

	AI = "Adenoma"	AI = "Non-Adenoma"
Change in agreement	OR = 3.17 [2.73, 3.70]	OR = 4.33 [3.70, 5.08]
Effect on confidence score	$\delta = 0.72$ [0.64, 0.80]	$\delta = -0.73$ [-0.79, -0.67]

Table B.4. Odds-Ratio (OR) for change in agreement and average effect δ on confidence score when the perceived AI response is "Adenoma" and "Non-Adenoma".

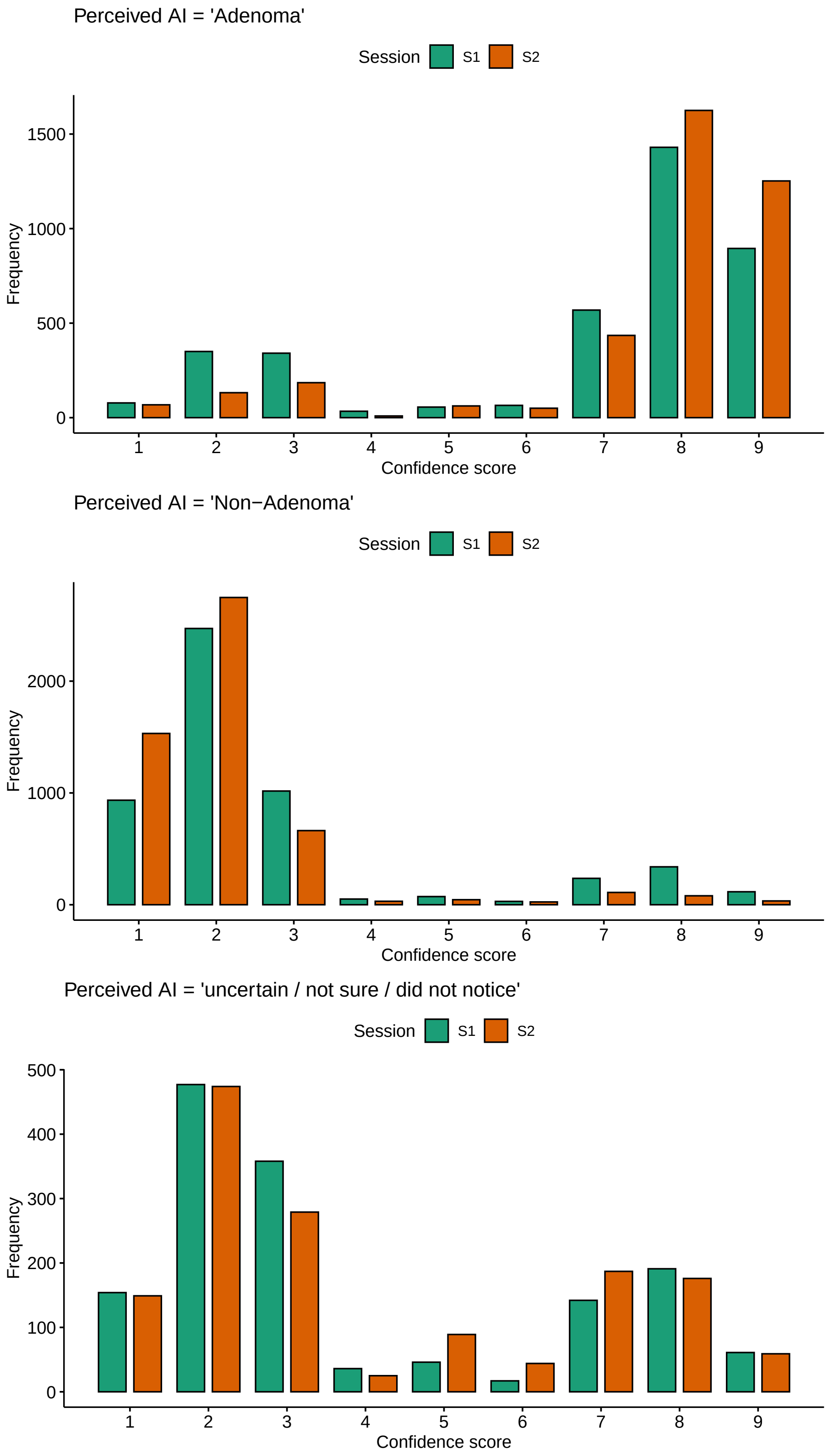

Figure B.12. Influence of the AI: confidence score frequency distribution in the two sessions S1 and S2 as a function of perceived AI response.

B.4 Visual determinants of AI perceived confidence

We explored what the visual determinants of the endoscopist perception of the AI confidence are. We consider two main explanatory variables:

1. Persistence, i.e., the highest number of consecutive frames in which the majority diagnosis (i.e., adenoma or non-adenoma) was shown, divided by the total number of frames with any AI output;

2. Proportion of 'No-prediction' frames in the AI output, i.e., the ratio between frames labeled 'No-prediction' and the number of frames where AI outputs 'Adenoma', 'Non-Adenoma' or 'No-prediction' (thus excluding 'Analyzing').

The 504 videos, each representing one lesion, are composed of 3285 frames on average (ranging from 575 to 16904). The lesion of interest was detected (i.e., the lesion is visible *and* the CADe highlights it with a box) on 1444 frames on average (ranging from 48 to 4859) displaying AI's dynamic output with labels 'Analyzing', 'Adenoma', 'Non-Adenoma' and 'No-prediction'. Fig. B.13 displays the relation between confidence of the AI, as evaluated by the endoscopists, persistence (left panel) and AI proportion of 'No-prediction'. Fig. B.14 shows the contour plot of persistence and proportion of 'No-prediction', separately by endoscopists' perceived AI confidence. NA corresponds to endoscopists' interpretations of the AI output of 'Uncertain' or 'I am not sure / I did not notice the output of the AI'.

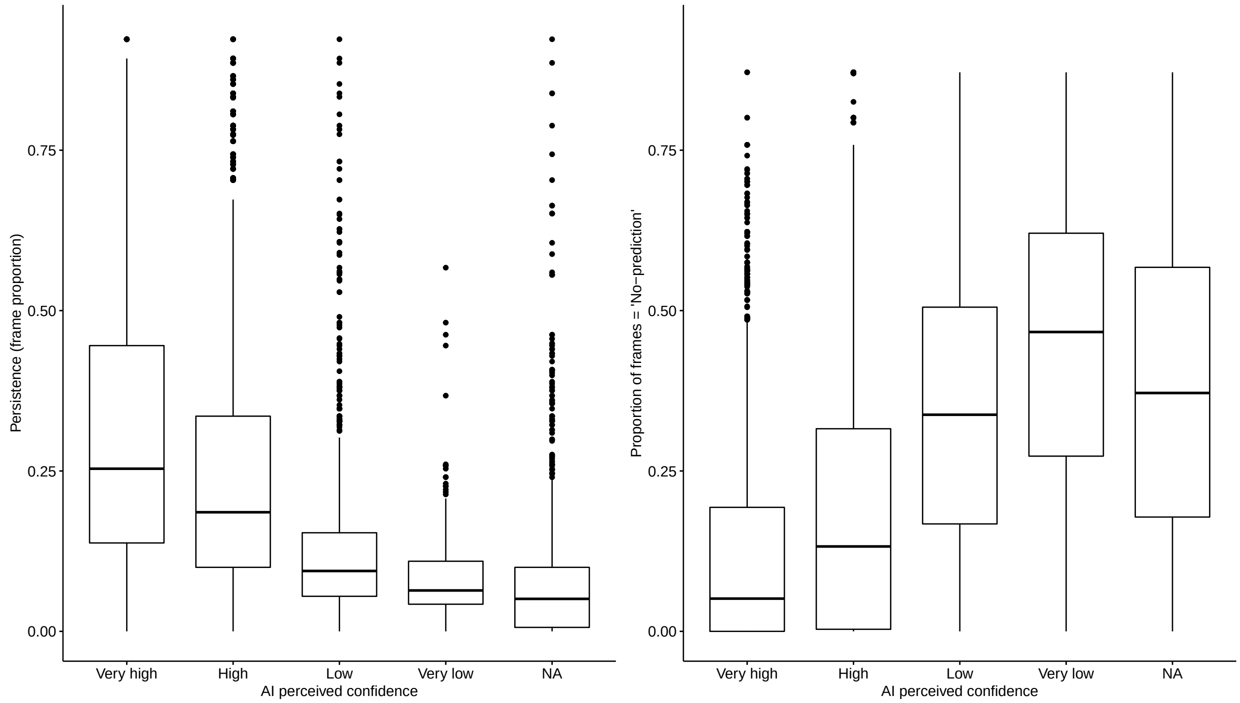

Figure B.13. Left Plot: Persistence as a function of endoscopists' perceived AI confidence. Right Plot: proportion of AI 'No-prediction' as a function of endoscopists' perceived AI confidence. NA corresponds to endoscopists' interpretation of the AI output as 'Uncertain' or 'I am not sure / I did not notice the output of the AI'.

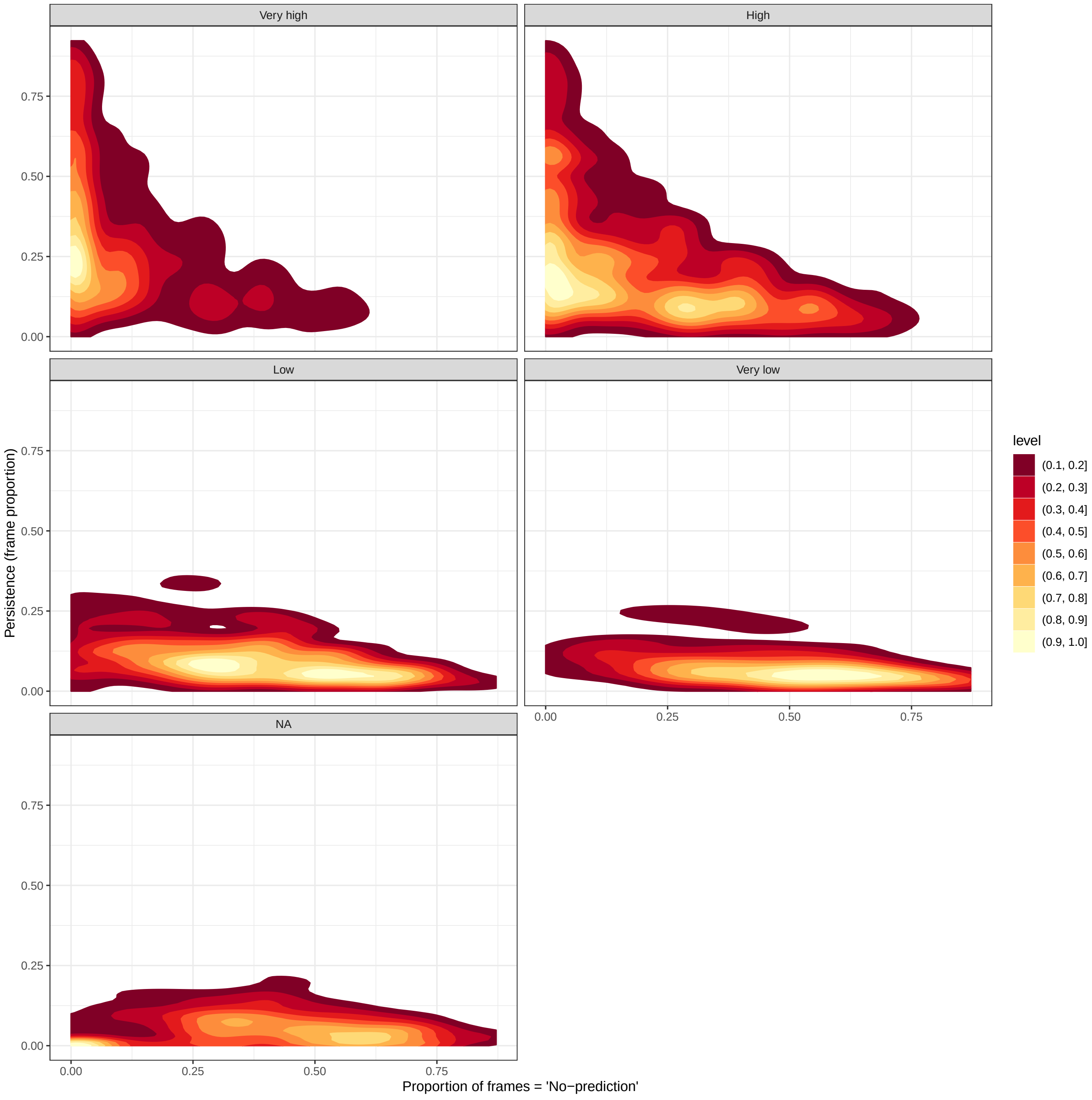

Figure B.14. Contour plot of Persistence and of the proportion of 'No-prediction' frames, separately by endoscopists' perceived AI confidence. NA corresponds to endoscopists' interpretation of the AI output as 'Uncertain' or 'I am not sure / I did not notice the output of the AI'.

	S1	S2	AI accuracy_s	AI accuracy_u
Expert	0.808	0.820	0.849	0.745
Non-expert	0.733	0.786	0.849	0.715

Table B.5. Raw accuracy in Session S1 and S2,and AI perceived correct diagnosis, separately by expertise of the endoscopists. Following the standard in the field, AI accuracy_s does not consider as wrong lesions where AI opinion was perceived as "uncertain" or was "not noticed". We also report the more conservative AI accuracy_u, which considers "uncertain" or "not noticed" as errors.

B.5 Detailed descriptive statistics

In this section we report detailed descriptive statistics for raw accuracy (Tab. B.5), specificity (Tab. B.6), sensitivity (Tab. B.7) and our main endpoints (OR for influence, accuracy, effectiveness and safety) separated for histologic classification (Tab. B.9).

	Spec. human (S1)	Spec. human (S2)	Spec. perceived AI_s	Spec. perceived AI_u
Expert	0.84	0.84	0.85	0.75
Non-expert	0.77	0.83	0.85	0.71

Table B.6. Specificity of human diagnosis (S1 and S2) and of AI perceived diagnosis. AI_s does not consider as wrong lesions where AI opinion was perceived as "uncertain" or was "not noticed". We also report the more conservative AI_u, which considers "uncertain" or "not noticed" as errors.

	Sens. human (S1)	Sens. human (S2)	Sens. perceived AI_s	Sens. perceived AI_u
Expert	0.75	0.79	0.84	0.74
Non-expert	0.67	0.72	0.85	0.72

Table B.7. Sensibility of human diagnosis (S1 and S2) and of AI perceived diagnosis. AI_s does not consider as wrong lesions where AI opinion was perceived as "uncertain" or was "not noticed". We also report the more conservative AI_u, which considers "uncertain" or "not noticed" as errors..

Receiver operating characteristic (ROC) have been widely used to evaluate the diagnostic performance in multireader, multicase (MRMC) studies, e.g. to compare AI and human experts for breast cancer detection in mammography[4].

In our experiment, multiple endoscopists (i.e., readers) evaluated multiple lesions (i.e., cases). For each lesion, each endoscopist rated his/her level of confidence towards the diagnosis (i.e., the "confidence score" ranging from 1 to 9, where 1 is "Very high confidence for Non-adenoma" and 9 is "Very high confidence for Adenoma"). The ground truth (i.e. "Adenoma" or "Non-adenoma") corresponded to the outcome of the histological evaluation.

The diagnostic performance of each endoscopist is described by a ROC curve showing the true positive proportion (Sensitivity) as a function of the false positive proportion (1 - Specificity), while the decision threshold varies. In the present case, the threshold is the specific confidence score that would be high enough for diagnosing an "adenoma". The ROC curve illustrates the trade-off between the sensitivity and the specificity of an endoscopist across all possible thresholds of the confidence score.

The area under an ROC curve (AUC) is a popular summary of the ROC curve, with higher AUC values indicate better performance, irrespective of a chosen threshold. Thus, the comparison in performance between S2 and S1 can be based on the difference between AUC-S2 (AUC of S2) and AUC-S1 (AUC of S1), with the assessment of the statistical significance of the difference taking into account the paired sample design.

Figure B.15 displays the ROC curves of all endoscopists, separately for S1 and S2. In 19 out of 21 endoscopists, AUC-S2 is larger than AUC-S1, indicating a better performance in S2 for most of the endoscopists. The sign test of symmetry between S1 and S2 gives a one-sided p-value of 0.0001. To describe the overall performance of the 21 endoscopists, Figure B.16 displays the ROC curve for each Session obtained by pooling individual confidence scores across endoscopists[5,6]. In the overall comparison, we see that S2 is superior to S1 since its ROC curve is higher everywhere, with AUC-S2$=0.847$ and AUC-S1$=0.809$. Figure B.17 shows the overall performance separately by expertise of the endoscopists (Expert vs Non-Expert). The AUC for S1 (experts) is 0.853; the AUC for S2 (experts) is 0.869.

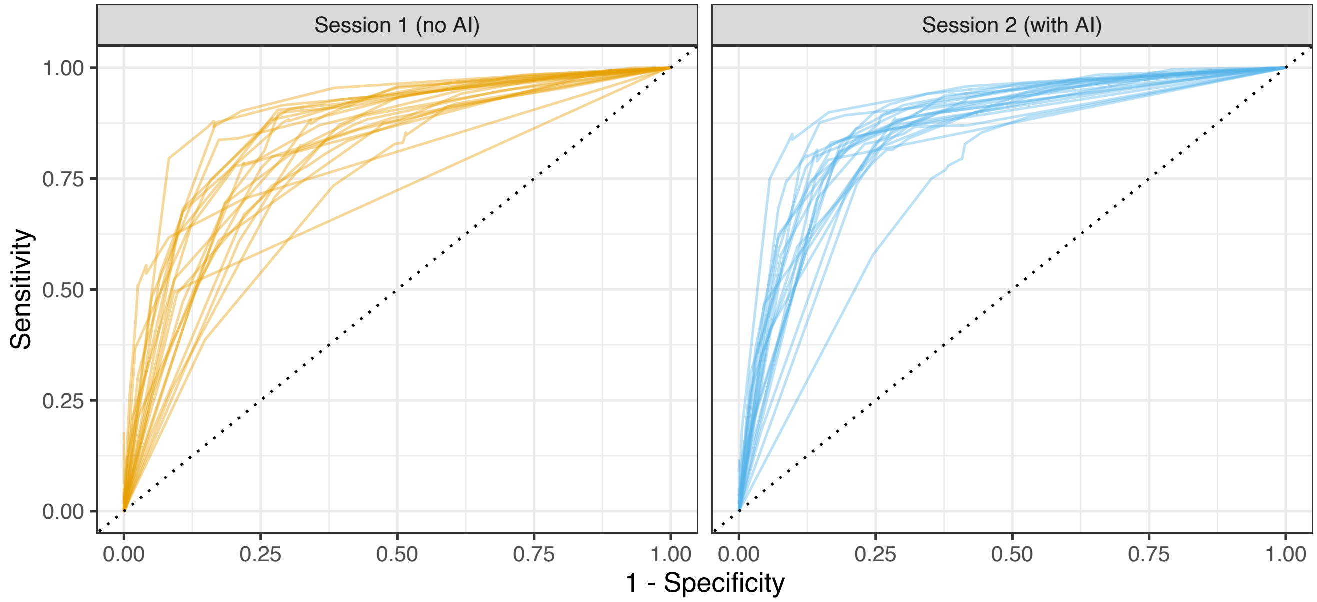

Figure B.15. ROC curves of a classification model in which "Histologic evaluation" is the binary output and "Confidence scores" are the dependent variables, for S1 and S2. The analysis is conducted separately for each endoscopist.

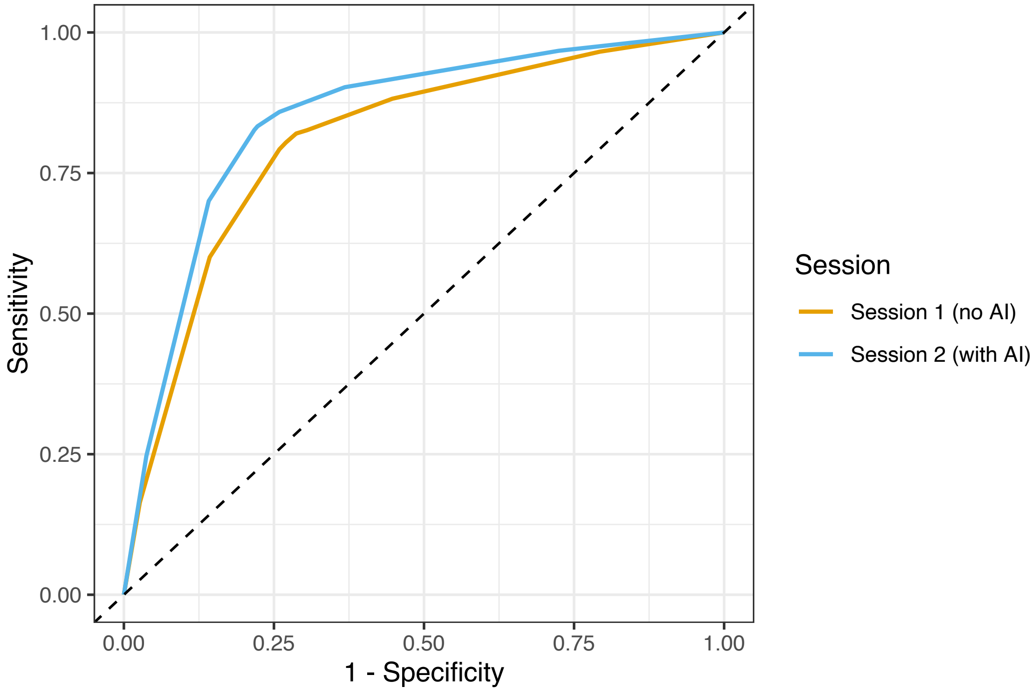

Figure B.16. ROC curves of a classification model in which "Histologic evaluation" is the binary output and "Confidence scores" are the dependent variables, for S1 and S2. The AUC for Session 1 is 0.809; the AUC for Session 2 is 0.847.

Confidence	Very low	Low	High	Very high
		Experts		
S1 accuracy	0.69	0.72	0.84	0.90
S2 accuracy	0.64	0.73	0.87	0.93
AI accuracy_s	0.68	0.71	0.87	0.93
		Non-experts		
S1 accuracy	0.59	0.62	0.75	0.81
S2 accuracy	0.58	0.63	0.83	0.89
AI accuracy_s	0.64	0.73	0.85	0.90

Table B.8. Proportions and sample sizes of the correct human diagnosis in S1, S2 and the AI perceived correct diagnosis, against different human confidence levels and the AI perceived confidence levels, respectively, for experts and non-experts. Accuracy_s does not consider as wrong lesions where AI opinion was perceived as "uncertain" or was "not noticed". Evaluations of the confidence of the AI were asked only when the opinion was "Adenoma" or "Non-Adenoma"

Endpoint	OR	Expert	Non-Expert
	Histology: adenoma		
1. Influence of the AI	ω_I	2.90 [2.32, 3.62]	3.04 [2.48, 3.72]
2. Diagnostic accuracy	ω_A	1.41 [1.17, 1.71]	1.52 [1.28, 1.81]
3. Effectiveness	ω_E	3.33 [2.48, 4.45]	3.25 [2.55, 4.14]
4. Safety	ω_S	0.62 [0.46, 0.83]	0.56 [0.43, 0.75]
	Histology: non-adenoma		
1. Influence of the AI	ω_I	2.94 [2.40, 3.60]	3.50 [2.91, 4.20]
2. Diagnostic accuracy	ω_A	0.98 [0.83, 1.16]	1.82 [1.56, 2.12]
3. Effectiveness	ω_E	3.60 [2.70, 4.80]	5.28 [4.15, 6.71]
4. Safety	ω_S	0.32 [0.25, 0.42]	0.63 [0.50, 0.80]

Table B.9. Odds-Ratios (OR) for each endpoint, estimated separately for experts and non experts and histology (adenoma and non-adenoma). We report in brackets the 95% confidence intervals.

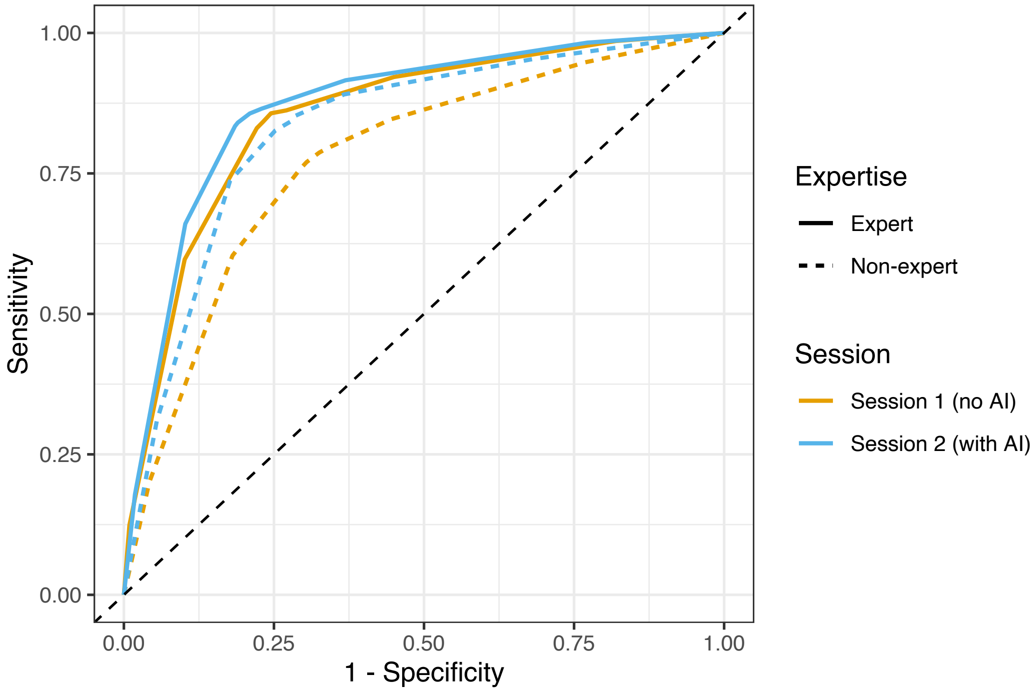

Figure B.17. ROC curves of a classification model in which “Histologic evaluation” is the binary output and “Confidence scores” are the dependent variables, for S1 and S2. The analysis is conducted separately for experts and non-experts. The AUC for Session 1 (experts) is 0.853; the AUC for Session 2 (experts) is 0.869. The AUC for Session 1 (non-experts) is 0.775; the AUC for Session 2 (non-experts) is 0.831.

References

1. Bates, D., Martin, M., Bolker, B. & Walker, S. Fitting linear mixed-effects models using lme4. *J. Stat. Softw.* **67**, 1–47 (2015).

2. Fay, M. P. Exact McNemar's test and matching confidence intervals (2011).

3. Dmitrienko, A. & D'Agostino Sr, R. Traditional multiplicity adjustment methods in clinical trials. *Stat. Medicine* **32**, 5172–5218 (2013).

4. Rodriguez-Ruiz, A. *et al.* Stand-alone artificial intelligence for breast cancer detection in mammography: comparison with 101 radiologists. *JNCI: J. Natl. Cancer Inst.* **111**, 916–922 (2019).

5. Metz, C. E. Some practical issues of experimental design and data analysis in radiological roc studies. *Investig. radiology* **24**, 234–245 (1989).

6. Chen, W. & Samuelson, F. W. The average receiver operating characteristic curve in multireader multicase imaging studies. *The Br. journal radiology* **87**, 20140016 (2014).
